# 1‐Alkali‐metal‐2‐alkyl‐1,2‐dihydropyridines: Soluble Hydride Surrogates for Catalytic Dehydrogenative Coupling and Hydroboration Applications

**DOI:** 10.1002/chem.201703609

**Published:** 2017-11-03

**Authors:** Ross McLellan, Alan R. Kennedy, Robert E. Mulvey, Samantha A. Orr, Stuart D. Robertson

**Affiliations:** ^1^ WestCHEM, Department of Pure and Applied Chemistry University of Strathclyde Glasgow G1 1XL UK

**Keywords:** catalysis, dehydrocoupling, hydroboration, lithium, main group

## Abstract

Equipped with excellent hydrocarbon solubility, the lithium hydride surrogate 1‐lithium‐2‐*tert*‐butyl‐1,2‐dihydropyridine (**1t**Li) functions as a precatalyst to convert Me_2_NH⋅BH_3_ to [NMe_2_BH_2_]_2_ (89 % conversion) under competitive conditions (2.5 mol %, 60 h, 80 °C, toluene solvent) to that of previously reported LiN(SiMe_3_)_2_. Sodium and potassium dihydropyridine congeners produce similar high yields of [NMe_2_BH_2_]_2_ but require longer times. Switching the solvent to pyridine induces a remarkable change in the dehydrocoupling product ratio, with (NMe_2_)_2_BH favoured over [NMe_2_BH_2_]_2_ (e.g., 94 %:2 % for **1t**Li). Demonstrating its versatility, precatalyst **1t**Li was also successful in promoting hydroboration reactions between pinacolborane and a selection of aldehydes and ketones. Most reactions gave near quantitative conversion to the hydroborated products in 15 minutes, though sterically demanding carbonyl substrates require longer times. The mechanisms of these rare examples of Group 1 metal‐catalysed processes are discussed.

## Introduction

**Figure 1:**
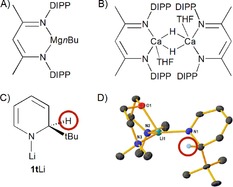
A) Depiction of DIPPnacnac‐Mg*n*Bu precatalyst; B) Depiction of (DIPPnacnac‐CaH⋅THF)_2_ precatalyst; C) 1‐Li‐2‐*tert‐*butyl‐1,2‐dihydropyridine unit; D) Molecular structure of **1t**Li⋅Me_4_AEE with all H atoms other than that bonded to the dihydropyridyl sp^3^ C atom omitted for clarity.7b

**Scheme 1:**
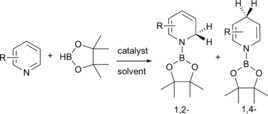
Depiction of catalytic hydroboration of pyridine.6b

The prevailing chemistry of dihydropyridines (DHPs) is dominated by their hydrogen‐transfer ability, a property resulting from their propensity to (re)gain the classic 6π electron aromaticity of the parent pyridine. The most important DHP is NADH (nicotinamide adenine dinucleotide), through its role in biology as an electron transporter used for energy creation.^1^ Two important examples of DHPs in synthetic chemistry are the Hantzsch esters,^2^ exploited, for example, under the name Nifedipine as calcium antagonists in hypertension treatment;^3^ and Lansbury's reagent Li^+^[Al(1,4‐NC_5_H_6_)_4_]^−^, a highly selective stoichiometric reducing agent.^4^ Hantzsch esters, and indeed most DHPs, exist as thermodynamically preferred 1,4‐isomers. With Lansbury's reagent, formed by reaction of LiAlH_4_ with excess pyridine, the isomeric ratio (1,2‐:1,4‐), and hence the active species identity in any given reaction is less clear and depends on reaction conditions, that is, the initially formed kinetic 1,2‐isomer converts to the 1,4‐isomer over time or with increased temperature.^5^ An emerging advance in the chemistry of main group (or d^0^) DHPs is the realisation of their usefulness in catalytic processes such as the hydroboration or hydrosilylation of pyridines and related heterocycles.^6^ Particularly noteworthy are reports by Hill who utilised a DIPPnacnac‐Mg*n*Bu (DIPPnacnac=[(2, 6‐*i*Pr_2_C_6_H_3_)NC(Me)]_2_CH) (Figure 1A) complex affording mixtures of 1,2‐ and 1,4‐DHP products, (Scheme 1)6a and Harder who used (DIPPnacnac‐CaH⋅THF)_2_ (Figure 1B) to selectively give 1,2‐DHP products.6d Significantly, each Group 2 catalysed reaction is proposed to involve M−H intermediates.

Recently we began to systematically investigate the synthesis and reactivity of a series of kinetically stable 1‐lithio‐2‐alkyl‐1,2‐dihydropyridines (**1**, Figure 1C) (alkyl=*n*‐, *i*‐, *s*‐ or *t*‐butyl) making the surprising finding that they can be isolated as stable solids provided that a 1:1 stoichiometric alkyllithium:pyridine ratio is used in their preparation.^7^ Significantly in the case of *s*‐, and *t*‐butyl isomers, **1s**Li and **1t**Li, the resulting cyclotrimeric aggregates were found to be soluble in hexane at room temperature, thus offering a synthetically important advantage over the insoluble rock salt lattice structure of LiH.^8^ **1t**Li can also be isolated as a monomer by coordination with neutral Lewis bases such as bis‐[2‐(*N,N*‐dimethylamino)ethyl]ether (Me_4_AEE) in **1t**Li⋅Me_4_AEE (Figure 1D). Promisingly, reactivity studies revealed that **1s**Li and **1t**Li are effective LiH transfer agents to the unsaturated C=O bond in benzophenone. Metathetical reactions of **1t**Li with NaO*t*Bu or KO*t*Bu resulted in the production of isolable heavier alkali‐metal congeners **1t**Na or **1t**K, both of which exhibit similar reactivity to **1t**Li in stoichiometric hydrometallation reactions.^9^ Moreover, we recently disclosed the first example of a Group 1 DHP complex (**1t**Li) functioning as an effective (pre)catalyst, in the dehydrogenative cyclisation of diamine boranes, therein establishing the dual role of 2‐*tert*‐butylpyridine as a LiH storage/release vessel,^10^ and moreover delivering a rare example of a lithium based precatalyst.

With a series of soluble alkali metal hydride surrogate congeners in hand we sought to examine their application in two distinct catalytic processes, namely dehydrocoupling of amine boranes and hydroboration of aldehydes and ketones. In each reaction metal hydride species have been found to either catalyse or have been identified as key intermediates in the process. The controlled formation of boron–nitrogen bonds by dehydrocoupling of amine boranes, HNR_2_⋅BH_3_ (R=H, alkyl) is a reaction that attracts widespread attention in the synthesis of novel polymers and ceramics,^11^ and in the arena of hydrogen storage materials.^12^ Thus over the past two decades, much activity has been directed at transition‐metal‐catalysed dehydrocoupling of ammonia borane and amine boranes, and moreover much insight has been garnered regarding mechanistic aspects of the various catalytic pathways.^13^ Recent insightful work from the groups of Harder,^14^ Hill^15^ and Wright,^16^ among others,^17^ demonstrated that main group (d^0^) complexes are active in both stoichiometric and catalytic dehydrocoupling of main group element–H bonds. Furthermore, Bertrand demonstrated that cross‐dehydrocoupling of secondary boranes with alcohols, thiols and amines can be accomplished without a catalyst.^18^ It is also noteworthy that precatalysts discussed in these reports tended to be more economically viable and environmentally innocuous than their invariably expensive and toxic noble transition metal counterparts, albeit at this point they do not (yet) match the best catalytic efficiencies. Among the most studied main group precatalysts are those from Group 2 and Group 13 which typically contain bulky β‐diketiminato or (silyl)amide ligands. Similarly these Group 2 complexes and related species have been found to catalyse the hydroboration of a range of substrates, including pyridines,^6^ aldehydes and ketones,^19^ nitriles,^20^ isonitriles,^21^ and esters.^22^ Impressively the hydrosilylation of alkenes using a potassium hydride catalyst was reported by Harder.^23^ More recently Okuda has provided mechanistic evidence for potassium catalysed hydrosilylation of a range of alkenes using a K(18‐crown‐6)(SiPh_3_) catalyst.^[24a ]^ Further, the Okuda group has recently demonstrated that alkali metal hydridotriphenylborates can catalyse the hydroboration of benzophenone.24b These transformations, for example converting an aldehyde into an alcohol, are of central importance within organic chemistry and have historically been accomplished using stoichiometric metal hydride species, for example LiAlH_4_, which can suffer from poor functional group selectivity and low solubility in hydrocarbon solvents.^25^ Thus utilisation of milder hydride sources (e.g., boranes) in tandem with a suitable catalyst remains a tantalising synthetic strategy. Breakthroughs reported herein will extend the versatility of hydrocarbon soluble Group 1 DHPs as metal hydride surrogates in the catalytic dehydrocoupling of amine boranes and in hydroboration of aldehydes and ketones. We also disclose the crucial importance of reaction solvent on catalytic efficiency.

## Results and Discussion

### Dehydrogenative coupling with a lithium dihydropyridyl precatalyst

**Figure 2:**
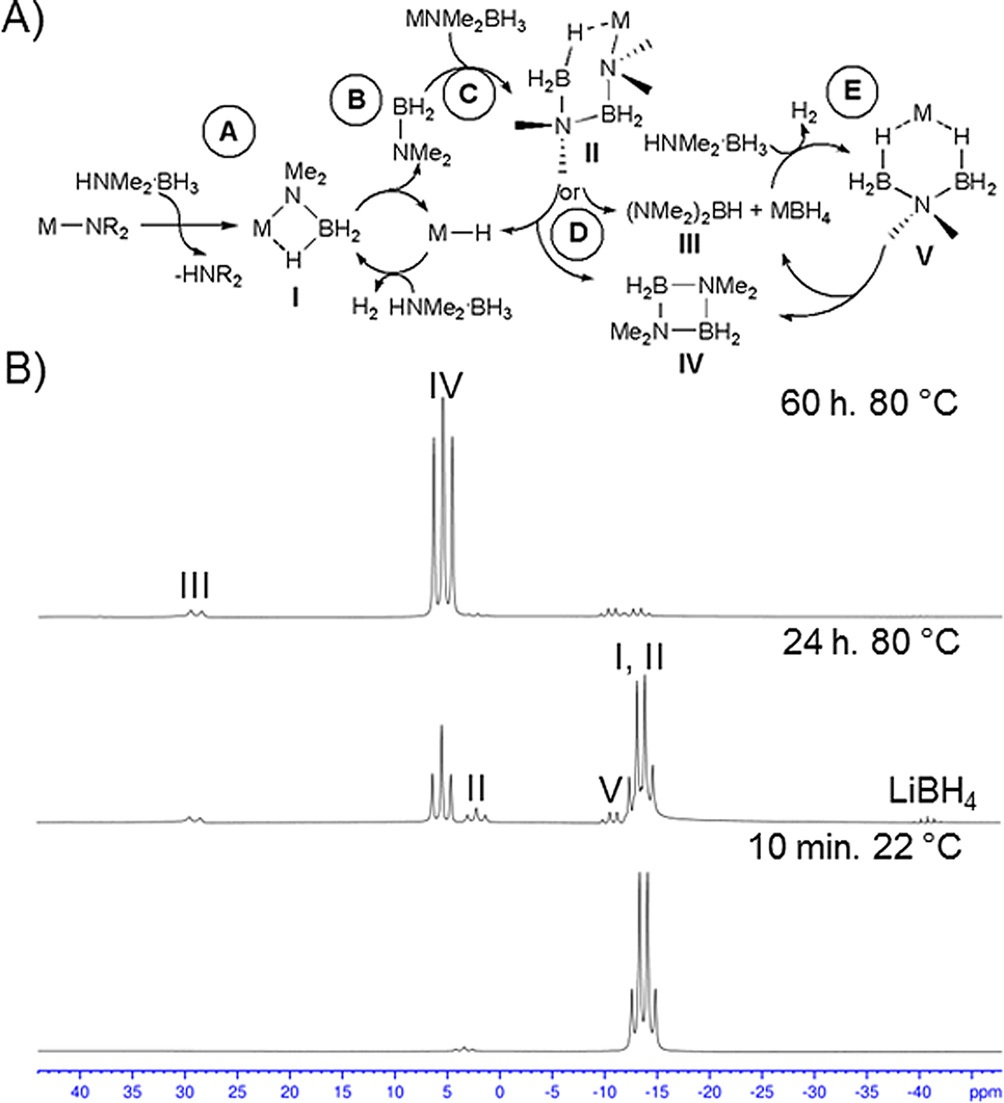
A) Proposed mechanism for d^0^‐based dehydrogenative coupling of HNMe_2_⋅BH_3_; B) ^11^B NMR spectra of the reaction between **1t**Li (2.5 mol %) and HNMe_2_⋅BH_3_ in [D_8_]toluene.

A particularly well understood substrate is dimethylamine borane, HNMe_2_⋅BH_3_, and a general mechanism has been proposed to rationalise its dehydrocoupling process15a (Figure 2A). Essentially the reaction follows four steps: A) metallation of HNMe_2_⋅BH_3_ by metal amide; B) β‐hydride elimination to afford a metal hydride and NMe_2_BH_2_; C) insertion of NMe_2_BH_2_ into another equivalent of the metallated amidoborane generated in step A (B−N bond forming step); D) β‐ or δ‐hydride elimination to afford final products, (NMe_2_)_2_BH (**III**) and (NMe_2_BH_2_)_2_ (**IV**), and regenerate metal hydride catalysts. Note step E is explained below. In certain cases intermediates containing a metal‐bound [NMe_2_BH_2_NMe_2_BH_3_]^−^ anion (**II**) were isolated and structurally characterised.15a,15b [NMe_2_BH_2_NMe_2_BH_3_]^−^ results from polar insertion of NMe_2_BH_2_ into M−NMe_2_BH_3_ and is the immediate precursor of the final reaction product(s). Complementary theoretical studies support this general mechanistic picture,^26^ though the β‐hydride elimination pathway (an apparent two‐step process) is reportedly energetically disfavoured.

Hill recently noted the first example of Group 1 silylamide precatalysts [MN(SiMe_3_)_2_, M=Li, Na, K] for dehydrocoupling of dimethylamine borane.^27^ 5 mol % of LiN(SiMe_3_)_2_ in toluene gave the best conversion, determined by ^11^B NMR, to 72 % [NMe_2_BH_2_]_2_ and 5 % (NMe_2_)_2_BH after heating at 80 °C for 124 h. In this study an intermediate potassium [NMe_2_BH_2_NMe_2_BH_3_]^−^ complex was isolated, indicating that the catalysis likely follows that suggested for Group 2 and 13 precatalysts (Figure 2A). These important results are more impressive given that the catalytically active metal hydride species are reported to form insoluble aggregates during the experiments, slowing down the process, particularly for the heavier alkali metal silylamides NaN(SiMe_3_)_2_ and KN(SiMe_3_)_2_. Solubility problems have also been encountered by Wright on employing LiAlH_4_ as a catalyst in a similar reaction with HNMe_2_⋅BH_3_, and by Panda in the LiN(SiMe_3_)_2_ catalysed cross‐dehydrocoupling of HBpin or 9‐BBN (9‐borabicyclo[3.3.1]nonane) with a range of amines, another rare example of Group 1 catalysis.^28^ It is therefore apparent that effective solubility of key metal hydrides is critical for high catalytic efficiency. Given that **1t**Li represents a soluble source of lithium hydride in hexane, we reasoned that the in situ generated metal hydride would exist as a soluble dihydropyridine species, thus enhancing the catalytic process.

**Scheme 2:**
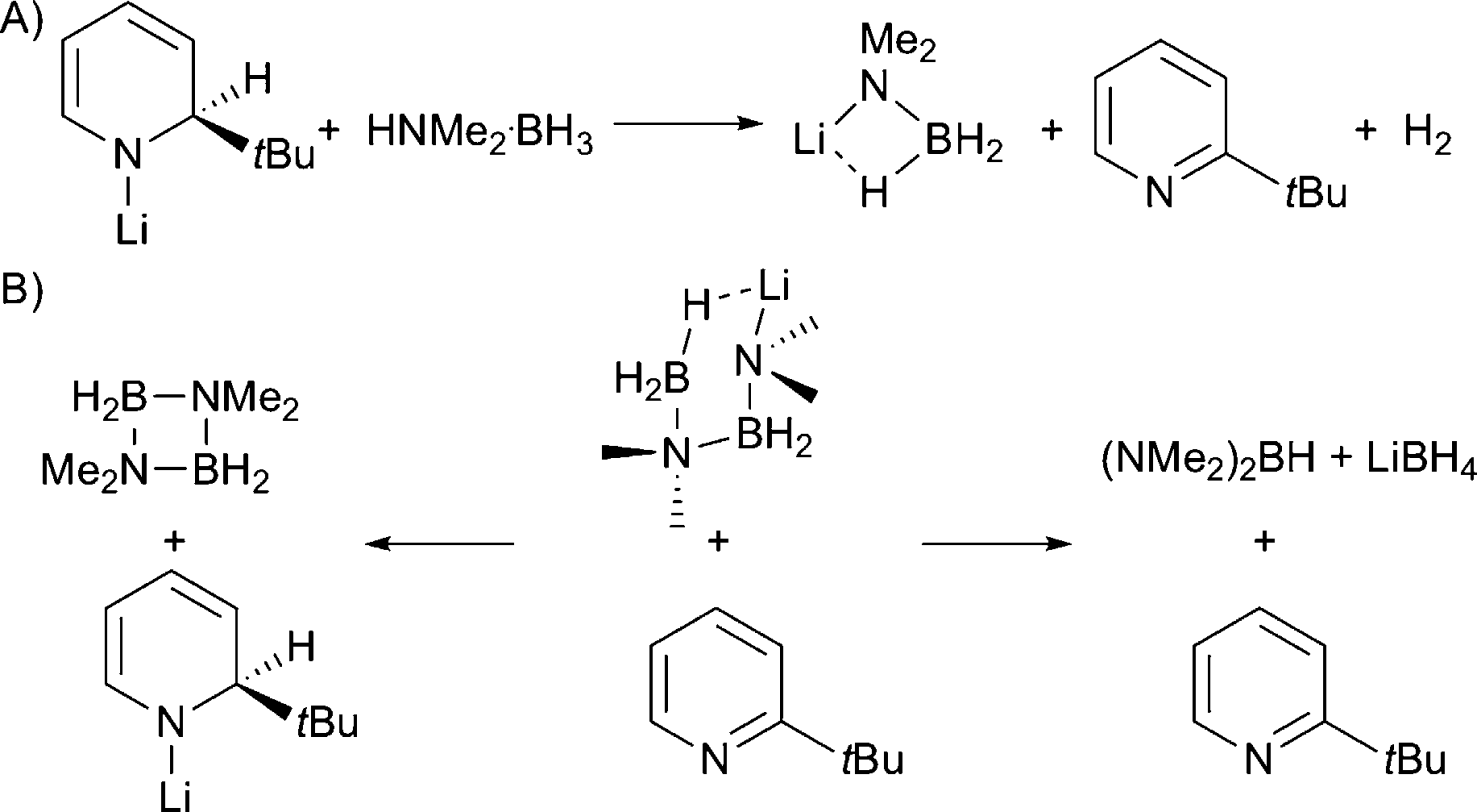
A) Proposed initial consumption of **1t**Li; B) Suggested formation of intermediate dihydropyridine (LHS: depicted here as 1,2‐DHP; but 1,4‐DHP or 1,6‐DHP isomers are also possible) from amidoborane intermediate and 2‐*tert*‐butylpyridine.

Reaction between 2.5 mol % **1t**Li and HNMe_2_⋅BH_3_ in [D_8_]toluene at 80 °C results in conversion (determined via ^11^B NMR integrals) to 89 % of [NMe_2_BH_2_] and 4 % of (NMe_2_)_2_BH after 60 h (Table 1 entry 2). Significantly this reaction proceeded faster than that of 5 mol % [Mg{CH(SiMe_3_)_2_}_2_(THF)_2_] with dimethylamine borane in [D_6_]benzene (72 h at 60 °C), indicating that **1t**Li is a competitive precatalyst.15a The in situ **1t**Li induced reaction was monitored by ^11^B NMR spectroscopy (Figure 2B) revealing the presence of several species (identified by comparison with literature data where appropriate). Initial mixing of the reagents in a J. Young's NMR tube resulted in immediate H_2_ gas evolution. This observation may be tentatively ascribed to the initial reaction between **1t**Li and HNMe_2_⋅BH_3_ forming Li[NMe_2_BH_3_] (**I**), 2‐*tert‐*butylpyridine and H_2_ (Scheme 2A). To be consistent with our hypothesis we expect that 2‐*tert*‐butylpyridine will act as a LiH storage/release vessel during the process, by forming dihydropyridines as a result of interaction with Li[amidoborane] species (Scheme 2B). At the initial time point the ^11^B NMR spectrum displays two resonances: a triplet at *δ*=3.4 ppm (^1^ *J*_BH_=100.1 Hz) corresponding to Li[NMe_2_ *BH_2_*NMe_2_BH_3_] (**II**)^27^ and a quartet composed of the mutually coincident signals16c, 27 of HNMe_2_ *BH_3_*, Li[NMe_2_ *BH_3_*] (**I**) and Li[NMe_2_BH_2_NMe_2_ *BH_3_*] (**II**) centred at *δ*=−13.6 ppm (^1^ *J*_BH_=96.2 Hz). The last named is formed by polar insertion of highly reactive NMe_2_BH_2_ into Li[NMe_2_BH_3_], in line with the literature mechanism. Analysis of the ^11^B NMR spectrum after heating the solution at 80 °C for 24 hours reveals the presence of several new species: a doublet at *δ*=28.9 ppm (^1^ *J*_BH_=129.9 Hz) confirmed as (NMe_2_)_2_ *BH* (**III**);^29^ a triplet at *δ*=5.4 ppm (^1^ *J*_BH_=113.1 Hz) assigned to cyclic dimer [NMe_2_ *BH_2_*]_2_ (**IV**);^30^ a partially obscured quartet centred around *δ*=−11.0 ppm (^1^ *J*_BH_=91.1 Hz) assigned to Li[NMe_2_(*BH_3_*)_2_] (**V**);16c and a quintet at *δ*=−40.9 ppm (^1^ *J*_BH_=81.0 Hz) corresponding to the borohydride Li[BH_4_].

**Scheme 3:**
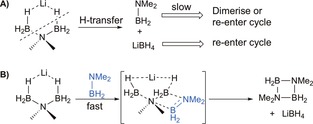
Proposed consumption of intermediate Li[NMe_2_(BH_3_)_2_] (**V**) by A) a hydride transfer, and/or B) polar insertion of NMe_2_BH_2_.

The emergence of Li[BH_4_] and Li[NMe_2_(BH_3_)_2_] (**V**) can be readily explained (step E Figure 2A). Borohydride Li[BH_4_] is the coproduct formed when the β‐hydride elimination pathway from Li[NMe_2_BH_2_NMe_2_BH_3_] is followed. Li[NMe_2_(BH_3_)_2_] is the result of deprotonation of HNMe_2_⋅BH_3_ by Li[BH_4_] and has been noted before by Wright, who rationally synthesized and structurally characterised the compound.16c As the reaction progresses it is apparent from ^11^B NMR data that the metallated amidoboranes are consumed. In the case of Li[NMe_2_BH_2_NMe_2_BH_3_] it is clear that the major process is δ‐hydride elimination to produce [NMe_2_BH_2_]_2_ (**IV**). We propose that Li[NMe_2_(BH_3_)_2_] is consumed via one (or both) of two similar routes. The first scenario involves a hydride transfer which would reform Li[BH_4_] and also generate BH_2_NMe_2_ (Scheme 3A). Both compounds could then re‐enter the catalytic cycle, or in the latter case an off‐metal dimerization pathway is conceivable. Alternatively, a molecule of NMe_2_BH_2_ could insert into Li[NMe_2_(BH_3_)_2_] giving [NMe_2_BH_2_]_2_ and Li[BH_4_] directly (Scheme 3B). Although a definitive pathway has not been discovered it is clear that Li[NMe_2_(BH_3_)_2_] is an important product‐forming intermediate in main group catalysed dehydrocoupling processes. A further important observation from ^11^B NMR data is that at high conversions to products, that is, low concentrations of HNMe_2_BH_3_/Li[NMe_2_BH_3_] a triplet of very low intensity is observed at *δ*=38.1 ppm (^1^ *J*_BH_=132.8 Hz) corresponding to NMe_2_BH_2_. The presence of this intermediate is somewhat surprising since it reacts/inserts very rapidly at early stages in the reaction. The inference is that the off‐metal dimerization step is likely to be very slow and thus insertion is preferred for NMe_2_BH_2_ giving credence to the amidoborane insertion path proposed in Scheme 3B. Altogether, the higher conversion, lower catalyst loading and shorter timescale found with **1t**Li, compared to the current state of the art, suggests that the presence of DHP species is important in the enhancement observed in these reactions.

### Dehydrogenative coupling with the sodium and potassium dihydropyridyl precatalysts

Next we assessed the role of alkali metal on the reaction. Sodium (**1t**Na) and potassium (**1t**K) variants were prepared via a simple and high yielding metathetical approach.^9^ Employing **1t**Na or **1t**K in catalytic reactions (Table 1 entries 3, 4) under analogous conditions used for **1t**Li resulted in similar conversions in both cases. All three reactions appear to proceed via similar routes since the analogous intermediates are observed in each case in the ^11^B NMR spectra (see Supporting Information). Notably these results compare very favourably with literature values (conversions to >85 % [NMe_2_BH_2_]_2_ with **1t**Na or **1t**K compared with approximately 43 % with NaN(SiMe_3_)_2_ or KN(SiMe_3_)_2_).^27^ Reaction timescales were comparatively long (72 h for **1t**Na and 144 h for **1t**K) with respect to **1t**Li (60 h), albeit considerably shorter than the reported values for NaN(SiMe_3_)_2_ and KN(SiMe_3_)_2_ (both 172 h). Thus, it seems clear that the issues with modest conversion in previous Na and K based catalysis, which was attributed to poorly soluble M−H species, has been somewhat resolved via use of “M−H solubilising” alkali metal alkyl‐dihydropyridine precatalysts. That **1t**Li outperforms the Na and K precatalysts agrees with both the enhanced solubility and the trend observed previously in main group dehydrocoupling systems,^27^ in which slower activity may be attributed to: increasing cation radius which promotes a longer, looser M⋅⋅⋅H−B contact and slows down hydride elimination; or the more dispersed charge density at the d^0^ metal which affects steps involving polar insertion of unsaturated fragments or σ‐bond metathesis leading to product formation.

The influence of reaction solvent was also investigated using **1t**Li as a representative precatalyst (Table 1 entries 5–8). Conducting the reaction in [D_12_]cyclohexane results in high conversion (94 %) to [NMe_2_BH_2_]_2_, albeit only after heating at 75 °C for 168 h. This comparatively long timescale is attributed to poor solubility of the dimethylamine borane starting material in cyclohexane slowing down the reaction. By moving to a more polar reaction medium, [D_8_]tetrahydrofuran, the reaction slowed considerably more, only reaching a conversion of 88 % [NMe_2_BH_2_]_2_ after 360 h. Presumably efficient stabilising Lewis base solvation of lithiated amidoboranes inhibits the polar insertion of NMe_2_BH_2_ into Li[NMe_2_BH_3_] and/or the hydride elimination steps. Moreover, it suggests that in this case fast catalytic turnover is reliant on the level of alkali‐metal solvation. The solvent effect here is in contrast to that reported by Wright,16c where both toluene and THF gave similar results with LiAlH_4_ as catalyst, albeit the poor solubility of LiAlH_4_ in hydrocarbon solvents may be a factor in this report. To assess the donor effect more thoroughly, the reaction was repeated with a donor solvated complex of **1t**Li in [D_8_]toluene, thereby differentiating any effect from bulk donor solvent (Table 1 entry 7). We selected previously reported chelate complex **1t**Li⋅Me_4_AEE,7b where two N and one O donor sites of the tridentate ligand fill three Li coordination sites. Reaction using **1t**Li⋅Me_4_AEE in toluene is faster than **1t**Li in bulk THF (120 vs. 360 h) although it is still much slower than unsolvated **1t**Li in toluene. Therefore it is clear that the level of solvation of the alkali metal is pivotal in this process.

**Table 1:** Catalytic conversion of HNMe_2_⋅BH_3_ to [Nme_2_BH_2_]_2_ (**IV**) and (Nme_2_)_2_BH (**III**) using DHP precatalysts.

	Precatalyst (mol %)	Deuterated solvent	*t* [h]	*T* [°C]	(**IV**) [%]	(**III**) [%]
**1**	**1**tLi (5 %)	toluene	72	80^[a]^	86	7
**2**	**1**tLi (2.5 %)	toluene	60	80	89	4
**3**	**1**tNa (2.5 %)	toluene	72	80	89	4
**4**	**1**tK (2.5 %)	toluene	144	80	86	8
**5**	**1**tLi (2.5 %)	C_6_D_12_	168	75	94	2
**6**	**1**tLi (2.5 %)	THF	360	65	88	4
**7**	**1**tLi⋅Me_4_AEE (2.5 %)	toluene	120	80	81	8
**8** ^[b]^	**1**tLi (2.5 %)	pyridine	5	80	2	94
**9** ^[b]^	2 (1.25 %)	pyridine	5	80	4	94
**10**	2 (1.25 %)	toluene	146	80	78	9
**11** ^[b]^	LiAlH_4_ (2.5 %)	pyridine	9	80	2	88
**12**	**1**tNa (2.5 %)	pyridine	8	80	2	91
**13**	**1**tK (2.5 %)	pyridine	7	80	<1	98

[a] Initial 24 h at 22 °C. [b] Resonance corresponding to III obscures a second reaction product, that increases with respect to III when heating is prolonged after consumption of HNMe_2_⋅BH_3_.

**Scheme 4:**
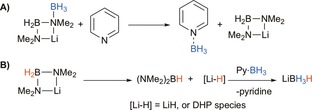
Alternative proposed reaction sequence accounting for the role of pyridine.

Surprisingly, moving to bulk pyridine (Table 1 entry 8) results in a remarkable acceleration of the reaction. Even more unexpected is the ratio of products dramatically switches such that near quantitative conversions of the diamine borane (94 % in 5 h) to (NMe_2_)_2_BH rather than [NMe_2_BH_2_]_2_ are obtained (note that since (NMe_2_)_2_BH is the major product, a stoichiometric quantity of boron remains unaccounted for by analysing the products observed in the ^11^B NMR spectrum. The identity of the “missing” boron has not been proven, however it is unlikely to be lost as B_2_H_6_, since diborane was not identified in NMR reaction monitoring). At this point it is unclear why the presence of bulk pyridine results in such a pronounced switch in reactivity. Analysis of ^11^B NMR data reveals the presence of Li[NMe_2_BH_2_NMe_2_BH_3_] (**II**) and Li[NMe_2_(BH_3_)_2_] (**V**), the same intermediates observed in the catalysis conducted in [D_8_]toluene, alongside an additional overlapping quartet resonance. Therefore, the main catalytic process may be considered to proceed via a similar route as in toluene, except that the product formation step is β‐H elimination from Li[NMe_2_BH_2_NMe_2_BH_3_] (vide supra), which can be tentatively explained by some pyridine “induced” change in charge polarisation over the intermediate, that is, coordination of pyridine to a boron atom in the intermediate would lead to a change in the charge distribution across the molecule. Sicilia previously disclosed that the in silico energetics of the (NMe_2_)_2_BH product forming steps are very high in energy for a related Mg^II^ system.^26^ Clearly the solvation effect of excess pyridine in some way promotes the hydride transfer from Li[NMe_2_BH_2_NMe_2_BH_3_] giving (NMe_2_)_2_BH. An alternative explanation for preferential (NMe_2_)_2_BH formation is that in a secondary competing process, a BH_3_ group is transferred to pyridine at some stage in the process forming the Py⋅BH_3_ adduct, which is in line with the additional low intensity quartet present in the ^11^B NMR spectrum. A control reaction of HNMe_2_⋅BH_3_ in [D_5_]pyridine at 80 °C for 20 h. confirms that BH_3_ transfer from HNMe_2_⋅BH_3_ to pyridine does not occur to any significant extent (ca. 15 % is present at *δ*=−11.2 ppm after prolonged heating). An alternative proposed reaction sequence; accounting for the unexpected reactivity in pyridine is given in Scheme 4.

**Scheme 5:**
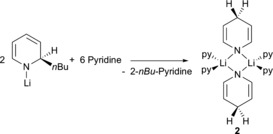
Synthesis of **2**.

The initial deprotonation and insertion steps remain the same. However, the intermediate Li[NMe_2_BH_2_NMe_2_BH_3_] has been depicted in an alternative conformation, ideally suited to transfer BH_3_ to a molecule of pyridine (Scheme 4A). From here, elimination of LiH (possibly as a dihydropyridine species), and reaction with the pyridine borane adduct would account for the formation of LiBH_4_ (Scheme 4B). It is also important to state that the identity of the precatalyst in pyridine solution is likely to be different from **1t**Li. Reaction of the *n*‐butyl isomer of **1t**Li with excess pyridine results in a 1,4‐dihydropyridyl bridged lithium dimer, [py_2_Li(−μ‐1,4‐DHP)]_2_ (**2**), with each Li atom solvated by two pyridine molecules (Scheme 5).^31^ Therefore it is likely that the active catalytic species more closely resembles **2** than **1t**Li. **2** was synthesised and tested as a precatalyst (1.25 mol %) in [D_5_]pyridine and in [D_8_]toluene (entries 9 and 10). In [D_5_]pyridine the reaction is complete in 5 hours, essentially replicating the reactivity observed using **1t**Li, reinforcing the idea that in pyridine **1t**Li converts to a species resembling **2**.

**Scheme 6:**
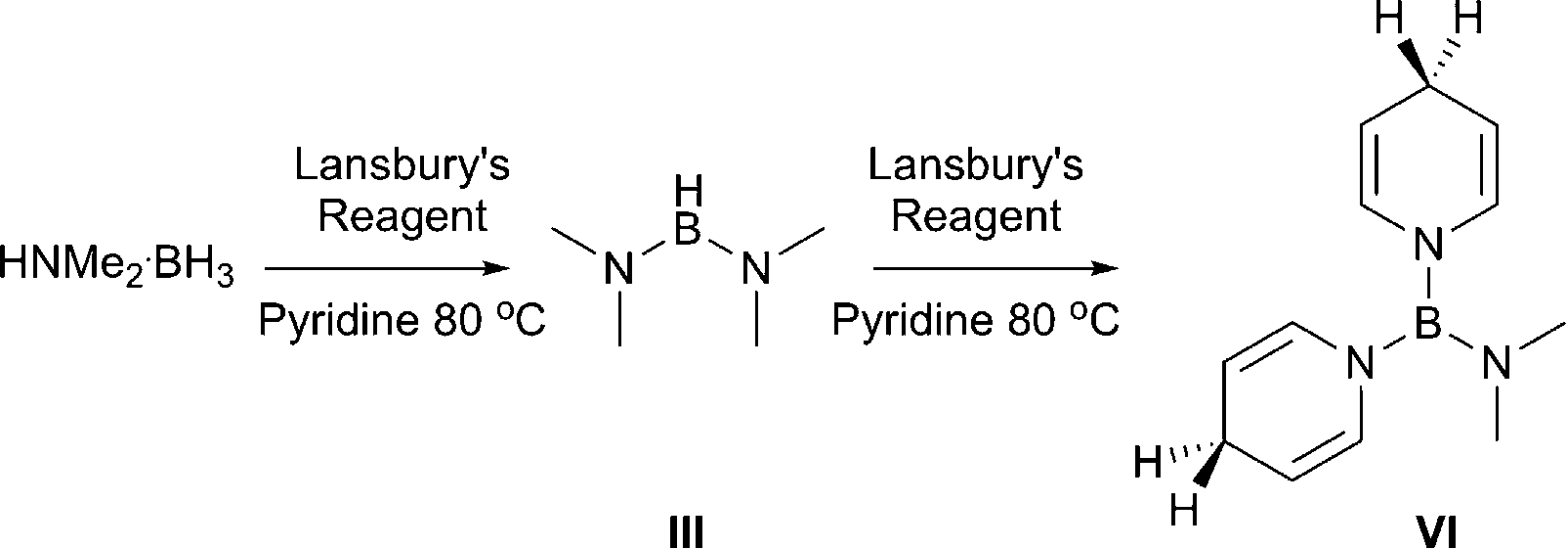
Synthesis of **VI**, formed by hydroboration with **III**.

In [D_8_]toluene the catalysis is much slower. Initially the product ratio is only approximately 3:1 in favour of **IV** over **III**, highlighting the influence of pyridine in product determination (here there are two equivalents of pyridine for each LiDHP). However, as the reaction proceeds the ratio changes to approximately 9:1 after 146 h. Exploring the concept of solvent control further we elected to employ LiAlH_4_ as a catalyst in [D_5_]pyridine (i.e., a catalytic amount of the usually stoichiometrically employed Lansbury's reagent). Further, Wright demonstrated that LiAlH_4_ is an effective catalyst in dehydrocoupling of dimethylamine borane in THF and toluene. Once more, the use of pyridine as reaction solvent results in high consumption of HNMe_2_⋅BH_3_, after 9 h at 80 °C, forming **III** as the major product (entry 11). Together these findings outline the importance of reaction solvent and suggest that a control of various dehydrocoupling reactions can be achieved with careful selection of precatalyst/solvent combinations. Interestingly, in each case where pyridine was used as a reaction solvent, prolonged heating of the reaction, after consumption of starting material results in the appearance of a partially obscured singlet resonance at about *δ*=26 ppm, alongside that corresponding to (NMe_2_)_2_BH in the ^11^B NMR spectra. The similarity of (NMe_2_)_2_BH to the commonly used hydroboration reagents pinacol or catechol borane, prompted us to consider whether, once formed, could then **III** hydroborate pyridine in the presence of a lithium DHP catalyst. A stoichiometric reaction between LiAlH_4_ and HNMe_2_⋅BH_3_ at 80 °C in bulk pyridine was conducted to test this hypothesis (Scheme 6). After removal of solvent, the crude solid, identified as primarily Lansbury's reagent, was washed with hexane and the hexane washings were subsequently analysed by NMR spectroscopy. Crucially the ^11^B NMR spectrum revealed the expected singlet at *δ*=26.4 ppm. The ^1^H NMR spectrum displayed three equal intensity multiplets at 5.96, 4.53 and 2.95 ppm, characteristic of a 1,4 dihydropyridine species. A singlet at 2.31 ppm can be assigned as the methyl hydrogens of an NMe_2_ group. The ratio of the peaks are in agreement with those of (DHP)_2_B(NMe_2_) (**VI**), indicating HNMe_2_ has been lost from **III** during the reaction.

Importantly this result indicates that a DHP based catalyst is still active in pyridine after expected product formation, and further, proving the hypothesis provided us with an impetus to test **1t**Li as a hydroboration precatalyst under more controlled conditions. Finalising our investigations in [D_5_]pyridine the reaction was repeated using precatalysts **1t**Na and **1t**K under analogous conditions (Table 1, entries 12 and 13). In both cases conversion of HNMe_2_⋅BH_3_ to (NMe_2_)_2_BH was rapid (ca >90 % in 8 h), albeit again slower than for **1t**Li, and interestingly the product resonances were clean with no presence of the hydroboration product.

### Hydroboration of aldehydes and ketones

Seeking to achieve our aim of extending the versatility of **1t**Li (the best performing precatalyst from the preceding section) in a catalytic regime we next attempted a series of hydroboration reactions with a selection of aldehydes and ketones using pinacol borane (HBpin). Traditionally HBpin is employed in hydroboration due to its hydridic hydrogen and electrophilic boron, however a recent break‐through has demonstrated it can also be employed as an easily accessed source of nucleophilic boron.^32^ These hydroboration products are important intermediates in the synthesis of alcohols from aldehydes and ketones, and remove the necessity to use a stoichiometric amount of metal reducing agent. Hill reported that DIPPnacnac‐Mg*n*Bu is an excellent precatalyst for this reaction, which proceeds with low catalyst loadings, high conversions and mild conditions.^19^ Moreover a Mg−H species was pinpointed as the active catalyst, involved in the first step of a two‐step process. The first step is insertion of the unsaturated carbonyl compound into the Mg−H bond. The second step, a metathesis with HBpin, affords hydroborated product and regenerates the active catalyst. We have already disclosed that alkali‐metal DHPs can efficiently transfer Li−H to benzophenone,^7^, ^9^ a reaction that mirrors the first step in the catalytic process since Li−H from **1t**Li adds across the C=O bond. Provided that the subsequent metathetical reaction with HBpin, in the presence of 2‐*tert*‐butylpyridine, regenerates an active 1‐lithio‐DHP then catalysis should proceed as described. Testing the hypothesis, benzaldehyde and HBpin were placed in a J. Young's NMR tube in [D_6_]benzene and the ^1^H and ^11^B NMR spectra were monitored over time after addition of 5 mol % **1t**Li. After 15 min at room temperature the ^1^H and ^11^B NMR spectra indicate essentially clean quantitative conversion to the hydroborated product, (Table 2, entry 1).

**Table 2:** Catalytic hydroboration of aldehydes and ketones using **1t**Li precatalyst in C_6_D_6_.

	Aldehyde/Ketone	*t* [h]	Yield as determined by ^1^H NMR [%]^[a]^
**1**	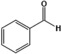	0.25	>99 [>95]^[c]^
**2**	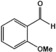	0.25	93
**3**	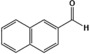	0.25	98
**4**	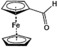	0.25	>99
**5**	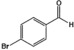	0.25	>99
**6** ^[b]^	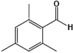	24	>99
**7**	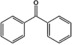	0.5	97
**8**	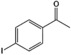	0.25	>99
**9**	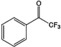	0.25	>99
**10**	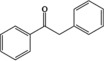	0.25	>99
**11**	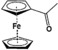	0.25	97
**12**	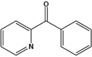	0.25	>98
**13** ^[b]^	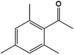	24	89
**14**	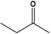	0.25	96
**15** ^[b]^	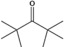	24	69

[a] Yield determined by formation of RR'CHOBpin relative to internal standard hexamethylcyclotrisiloxane. [b] Heated at 70 °C. [c] 1 % catalyst loading.

**Scheme 7:**
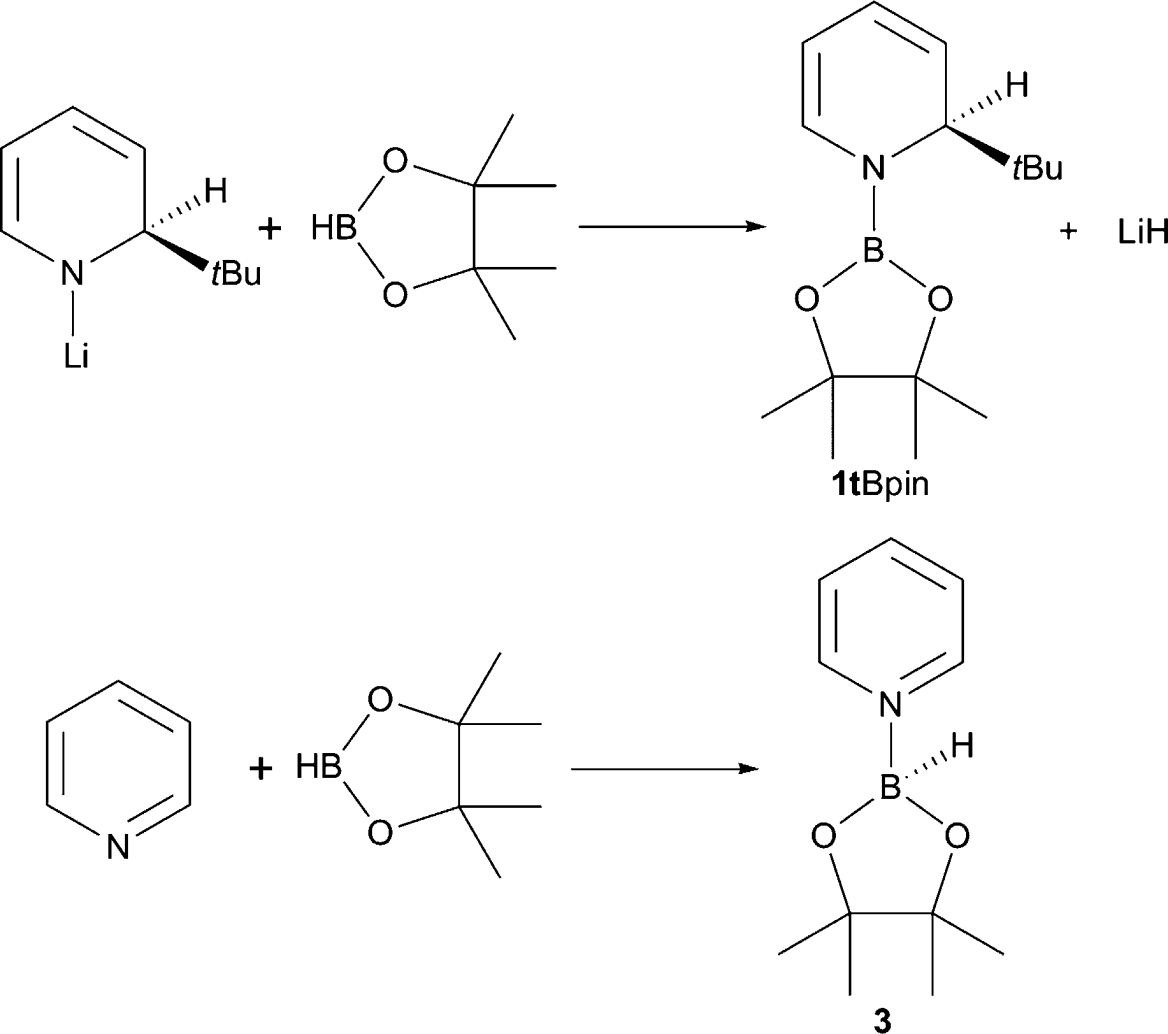
Control reaction of A) **1t**Li with HBpin giving **1t**Bpin and B) Pyridine with HBpin giving **3**.

**Figure 3:**
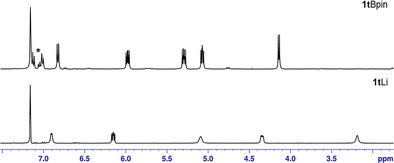
^1^H NMR spectra (dihydropyridyl region) of the reaction between **1t**Li and HBpin in [D_6_]benzene showing formation of **1t**Bpin. *=toluene.

Importantly the result demonstrates the versatility of Group 1 DHP based precatalysts since they can effectively catalyse both dehydrocoupling and hydroboration reactions. Next we turned our attention to extending the scope of aldehydes and ketones employed in hydroboration reactions using the same conditions. 2‐Methoxybenzaldehyde, 2‐naphthaldehyde and ferrocene carboxaldehyde (entries 2–4) are all cleanly converted into the corresponding protected alcohols after only 15 min at room temperature in high NMR yields (ca. 95 %) versus an internal standard. Notably the analogous reaction of 2‐methoxybenzaldehyde using DIPPnacnac‐Mg*n*Bu (0.5 mol %) is complete in one hour.^19^ Further, the hydroboration of 2‐naphthaldehyde is faster than that catalysed by the ruthenium complex [Ru(*p*‐cymene)Cl_2_]_2_ (0.1 mol %, 4 h),^33^ albeit lower catalyst loadings were used in each case. Hydroboration of 4‐bromobenzaldehyde (entry 5) is also complete within 15 min, indicating a tolerance to Li/halogen exchange under the reaction conditions, thereby increasing the range of useful substrates able to participate in these reactions. Furthermore this reaction occurs quicker than those using either 0.05 mol % Ar*N(Si(*i*Pr)_3_)SnO*t*Bu,^34^ in 4.5 h (Ar*=(C_6_H_2_{C(H)Ph_2_}_2_ *i*Pr‐2,6,4), (IPr)CuO*t*Bu,^35^ (0.1 mol %, 1 h) or [Ru(*p*‐cymene)Cl_2_]_2_ (0.1 mol %, 3 h), although again **1t**Li has a higher loading (5 mol %).^33^ Interestingly, hydroboration of mesitaldehyde (entry 6) takes longer for complete conversion (24 h at 70 °C). We attribute this to the steric hindrance of two *ortho*‐mesityl methyl groups, which slows down the process, presumably by either inhibiting the hydrometallation step and/or by preventing efficient reformation of the putative active DHP catalyst. Moving to ketones, the hydroboration potential of **1t**Li was examined with benzophenone as substrate (entry 7). Under the same conditions outlined above, clean conversion was achieved albeit after 30 min at room temperature. 4‐Iodoacetophenone and trifluoroacetophenone (entries 8 and 9) both react in high yields and with short reaction times (ca. >95 % in 15 min). In the latter case, Jones reports Ar*N(Si(*i*Pr)_3_)GeO*t*Bu (2.5 mol %, 15 min) and Ar*N(Si(*i*Pr)_3_)SnO*t*Bu (0.5 mol %, <15 min) precatalysts that perform the reaction with lower loadings or are slightly faster in the Sn case.^34^ Hydroboration of 2‐phenylacetophenone, 2‐acetylferrocene and 2‐benzoylpyridine (entries 10–12) are also complete in 15 minutes at room temperature, with in the third case efficient hydroboration occurring only at the carbonyl functionality. Once more the increased sterics of a mesityl substituted carbonyl (entry 13) necessitates a longer reaction (24 hours) and increased temperature (70 °C) to achieve full conversion. Dialkylketones are smoothly hydroborated, with 2‐butanone taking 15 minutes at room temperature (entry 14). Like the aryl systems, increased steric bulk necessitates longer times and higher temperatures, with di‐*tert*‐butyl ketone requiring 24 h at 70 °C to give almost 70 % conversion (entry 15). To assess whether the reaction may proceed via an alternative reaction pathway to that postulated for other main group systems (vide supra)^19^ a series of control reactions were performed. As dihydropyridines and their parent aromatic counterparts would be present in the reaction mixture, the reactivity between HBpin and **1t**Li and with pyridine (as a model variant of 2‐*tert*‐butylpyridine) were probed. The stoichiometric reaction between HBpin and **1t**Li in toluene at room temperature (Scheme 7 A) results in complete trans‐elementation giving in situ generated **1t**Bpin as evidenced by ^1^H NMR studies (Figure 3). Here the five proton resonances from the dihydropyridyl ring **1t**Li are replaced by five new dihydropyridyl resonances, consistent with replacement of lithium with a Bpin unit and presumably generating LiH as a coproduct. Furthermore the ^11^B NMR displays a singlet resonance at *δ*=24.5 ppm corresponding to the newly installed B−N bond.

Potentially **1t**Bpin could act as an active catalytic entity in the hydroboration process, therefore benzophenone was added to a reaction mixture containing **1t**Bpin to investigate whether it would convert to hydroborated product, and the reaction was monitored by ^11^B NMR spectroscopy. The emergence of a singlet at *δ*=23 ppm corresponds to the hydroborated product. For **1t**Bpin to act as a viable catalytic intermediate, conversion of the parent pyridine into a dihydropyridine species must occur by some mechanism. It is long established that commercial LiH, owing to its insolubility in organic media (originating from its considerable lattice energy), on its own does not add across pyridine, indicating this pathway is unlikely, albeit in situ generated LiH may exhibit higher reactivity in this regard.^31^ A second possibility is the direct addition of HBpin across the parent pyridine.

**Figure 4:**
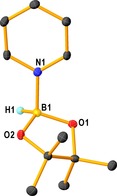
Molecular structure of **3**. Hydrogen atoms other than that attached to boron are omitted for clarity. Thermal ellipsoids are drawn at 30 % probability. Selected bond lengths (Å) and angles (°): B1−N1 1.651(2); B1−O1 1.442(2); B1−O2 1.452(2); B1−H1 1.164(18); N1‐B1‐O1 107.76(13); N1‐B1‐O2 108.41(13); N1‐B1‐H1 103.4(9); O1‐B1‐O2 107.45(15); O1‐B1‐H1 116.7(9); O2‐B1‐H1 112.6(9).

Direct reaction between HBpin and pyridine (Scheme 7B) suggests that hydroboration and concomitant dearomatisation of the pyridine does not readily occur. This was duly confirmed with an X‐ray crystallographic study, revealing the major product as the simple donor–acceptor adduct HBpin⋅py (**3**) in a 58 % yield. This structure represents the ‘pyridine‐activated HBpin’ intermediate postulated by Wright and co‐workers in their very recently reported boronium cation initiated hydroboration of pyridine.^36^ In **3**, B1 is in a distorted tetrahedral geometry [range of angles 103.4(9)–116.7(9)°] with respect to N1, O1, O2 and H1 (which was located and refined crystallographically, Figure 4). A search of the Cambridge Structural Database (CSD) surprisingly resulted in zero hits for HB(O)_2_ units bonded to pyridine. Crystals of **3** appear to decompose into a colourless oil after storage in an inert atmosphere glovebox. ^11^B NMR studies of the decomposition product reveal that as expected the major resonance is that of **3**, a doublet at *δ*=28.3 ppm accounting for about 80 % of the material via integration of the boron NMR spectrum. The remainder of the material is represented by a singlet at *δ*=23.9 ppm indicating a minor amount of hydroborated pyridine. In agreement the ^1^H NMR displays resonances potentially attributable to a DHP species, alongside the expected HBpin and pyridine resonances.

**Scheme 8:**
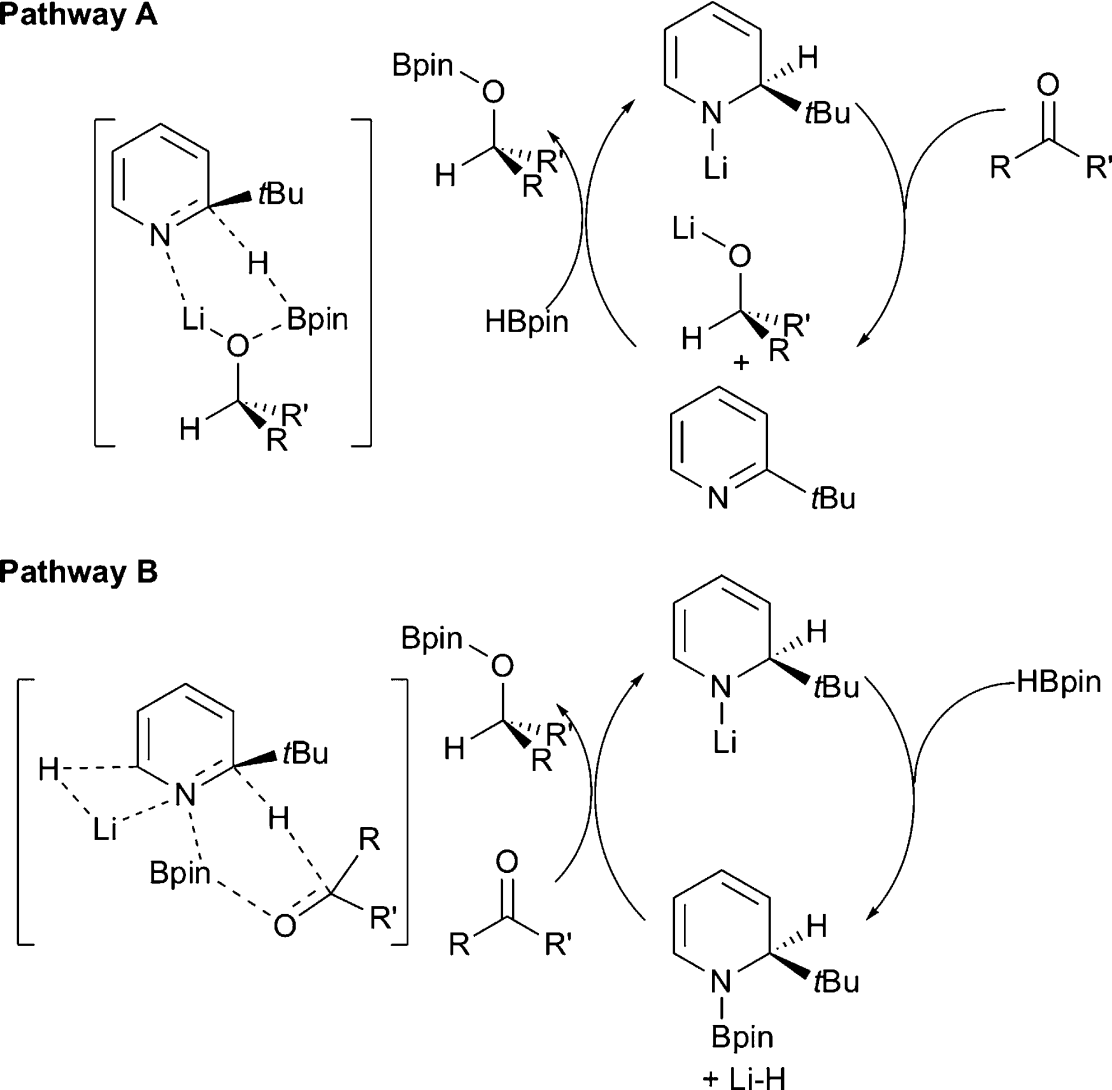
Proposed catalytic pathways A and B with hypothetical transition states for hydroboration of aldehydes and ketones with **1t**Li as precatalyst. DHP species are depicted as 1,2‐isomers; other isomers (1,4‐ or 1,6‐) are also possible.

Scheme 8 displays two potential routes for catalysis to proceed. Pathway A follows one commonly accepted mechanism of main group hydroboration catalysis (insertion/metathesis),^19^ albeit in this case pyridine/dihydropyridine plays an active role as a metal hydride storage/release vehicle. Alternatively pathway B describes a concerted process between **1t**Bpin, the carbonyl substrate, and the in situ generated LiH, explaining both hydroboration and catalyst reformation. It may be significant that in pathway B, LiH is generated in a step prior to aromatic pyridine formation. Due to the poor hydrocarbon solubility of LiH, polymeric LiH aggregates are likely to precipitate. Therefore one may expect pathway A to be the favoured catalytic manifold since LiH is generated in the presence of the aromatic pyridine and can therefore add across it in this regime. A second consideration in pathway B is that the incipient LiH may simply associate with excess HBpin giving a substituted borohydride species of the form Li[H_2_Bpin], and thereby remaining solubilized. However we see no spectroscopic evidence to support such a scenario.

## Conclusions

In conclusion, this study showcases the benefits of making molecular modifications of the classical salt lattice structures of the alkali metal hydrides. Dispensing metal hydrides in the form of molecular alkyl‐dihydropyridines has a profound positive impact on the dehydrogenative coupling of dimethylamine borane. Excellent hydrocarbon solubility of these alkali metal dihydropyridines and presumably of the metal hydride intermediates involved in the catalysis, are almost certainly key factors in the successful dehydrocoupling reactions. The usefulness of the lithium *tert*‐butyl‐dihydropyridine as a precatalyst was extended to pinacolborane sourced hydroboration reactions with a range of aldehydes and ketones. These catalytic applications demonstrate rare examples of group one based pre‐catalysts that advance the growing body of recent literature demonstrating that main group metal systems can in certain cases be successful in catalytic reactions previously thought to be the exclusive domain of transition metal systems. Future work will focus on just how far this analogy can be extended for these remarkable soluble hydride surrogates.

## **Experimental Section**

Full details of experimental procedures are provided in the electronic Supporting Information.

## Conflict of interest

The authors declare no conflict of interest.

## Supporting information

As a service to our authors and readers, this journal provides supporting information supplied by the authors. Such materials are peer reviewed and may be re‐organized for online delivery, but are not copy‐edited or typeset. Technical support issues arising from supporting information (other than missing files) should be addressed to the authors.

Supplementary
