# Supplementary material for: 1‐Alkali‐metal‐2‐alkyl‐1,2‐dihydropyridines: Soluble Hydride Surrogates for Catalytic Dehydrogenative Coupling and Hydroboration Applications
Source: Chemistry. 2017 Nov 3;23(66):16853–61. doi: 10.1002/chem.201703609 (PMC5820744; doi:10.1002/chem.201703609)
Supplement: Supplementary file 1 — Supplementary [file CHEM-23-16853-s001.pdf]

CHEMISTRY
A European Journal

Supporting Information

1-Alkali-metal-2-alkyl-1,2-dihydropyridines: Soluble Hydride Surrogates for Catalytic Dehydrogenative Coupling and Hydroboration Applications

Ross McLellan, Alan R. Kennedy, Robert E. Mulvey,* Samantha A. Orr, and Stuart D. Robertson*[a]

chem_201703609_sm_miscellaneous_information.pdf

1-Alkali-metal-2-alkyl-1,2-dihydropyridines: soluble hydride surrogates for catalytic dehydrogenative coupling and hydroboration applications

Ross McLellan,[a] Alan R. Kennedy,[a] Robert E. Mulvey,[a] Samantha A. Orr[a] and Stuart D. Robertson[a]

WestCHEM, Department of Pure and Applied Chemistry, University of Strathclyde, Glasgow, G1 1XL, UK

General experimental considerations

All reactions and manipulations were conducted under a protective argon atmosphere using either standard Schlenk techniques or an MBraun glove box fitted with a gas purification and recirculation unit. NMR experiments were conducted in J. Youngs tubes oven dried and flushed with Argon prior to use. Solvents were dried by heating to reflux over sodium benzophenone ketyl and then distilled under nitrogen prior to use. All other reagents were purchased commercially from Sigma-Aldrich and used as received. **1t**Li,[1] **1t**Na,[2] **1t**K[2] and **2**[3] were prepared as previously described or by slight variations thereof.

NMR Spectroscopy NMR spectra were recorded on a Bruker AV3 or AV 400 MHz spectrometer operating at 400.13 MHz for ^{1}H, 128.38 MHz for ^{11}B, 155.47 MHz for ^{7}Li and 100.62 MHz for ^{13}C. All ^{13}C spectra were proton decoupled. ^{1}H and ^{13}C NMR spectra were referenced against the appropriate solvent signal. ^{7}Li NMR spectra were referenced against LiCl in D_2O at 0.00 ppm and ^{11}B spectra were reference against $BF_3{\cdot}OEt_2$ in $CDCl_3$ at 0.00 ppm

X-ray Crystallography Crystallographic data were collected on Oxford Diffraction instruments with Mo Kα radiation (λ = 0.71073 Å). Structures were solved using SHELXS-97[4] or OLEX2,[5] while refinement was carried out on F2 against all independent reflections by the full matrix least-squares method using the SHELXL-97 program or by the GaussNewton algorithm using OLEX2. All non-hydrogen atoms were refined using anisotropic thermal parameters. Selected crystallographic details and refinement details are provided in table S1. CCDC 1551225 contains the supplementary crystallographic data for this structure. These data can be obtained free of charge from the Cambridge Crystallographic Data Centre via www.ccdc.cam.ac.uk/data_request/cif.

Catalytic dehydrocoupling procedure of dimethylamine borane with 2.5 mol% 1tLi

Dimethylamineborane (59 mg, 1 mmol) and **1t**Li (3.6 mg 2.5 mol%) were placed in a J. Youngs NMR tube and dissolved in the desired deuterated solvent. The NMR tube was then heated for the prescribed period and the reaction monitored via ^{1}H, and ^{11}B spectroscopy.

The same procedure was used for **1t**Li·AEE (7.6 mg 2.5 mol%), **1t**Na (4.0 mg 2.5 mol%), **1t**K (4.4 mg 2.5 mol%), **2** (6.1 mg, 1.25 %) and $LiAlH_4$ (1.0 mg, 2.5 mol%).

Catalytic hydroboration procedure

In a typical procedure the required substrate (0.5 mmol) was added to a J. Young NMR tube and dissolved in C_6D_6 (0.5 mL) containing 10 mol% of the internal reference standard hexamethylcyclotrisiloxane and the NMR data recorded. HBPin (0.076 mL, 0.5 mmol) and then **1t**Li (3.6 mg, 5 mol%) were added and the reaction monitored by NMR spectroscopy.

Figure S1: Catalytic dehydrocoupling of dimethylamine borane with **1t**Li in d_8-toluene (2.5 mol%) over 60 h. at 80 °C.

^{11}B NMR spectra

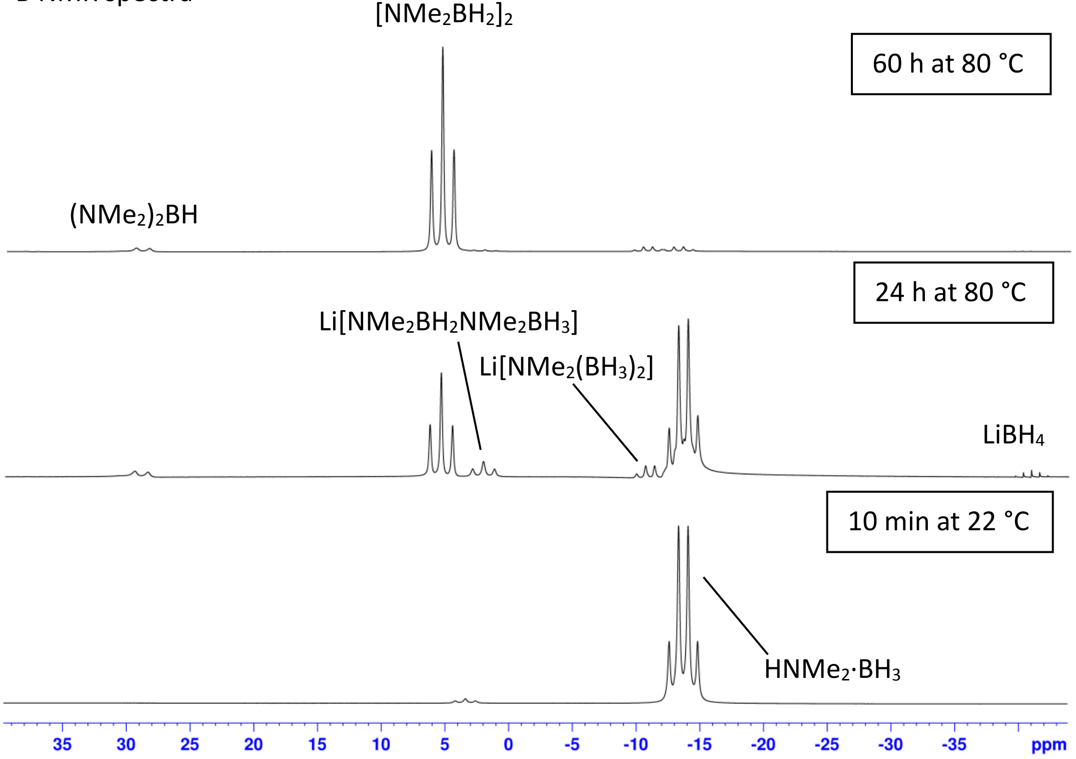

^{1}H NMR Spectra

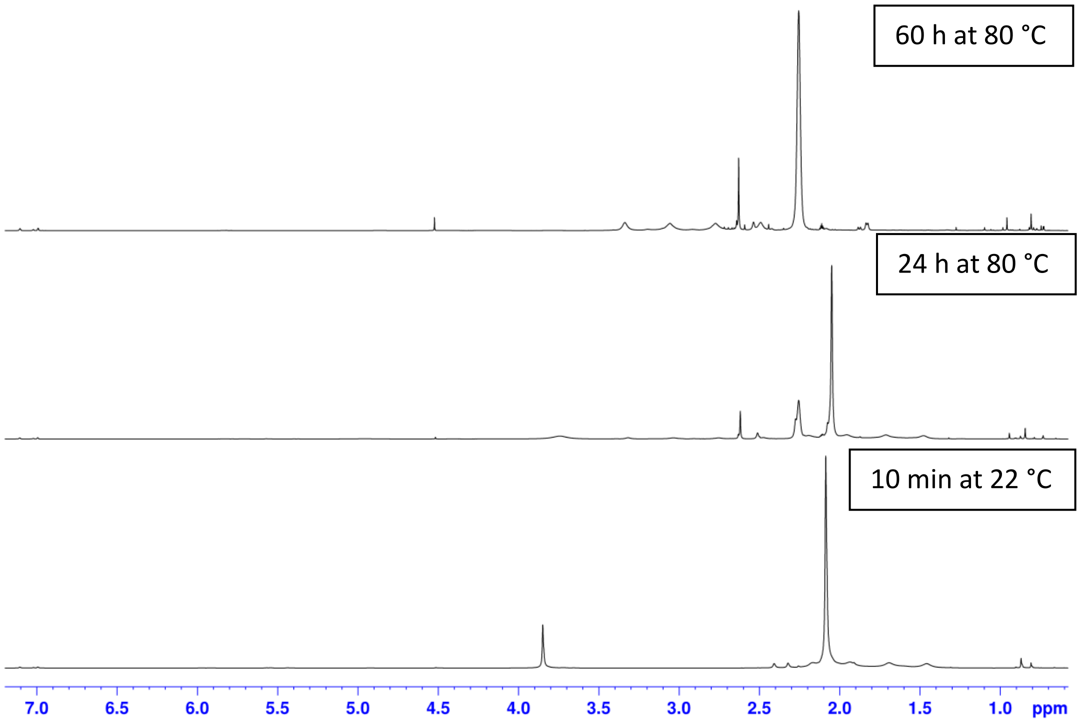

Figure S2: Catalytic dehydrocoupling of dimethylamine borane with **1t**Na (2.5 mol%) in d_8-toluene over 72 h. at 80 °C.

^{11}B NMR spectra

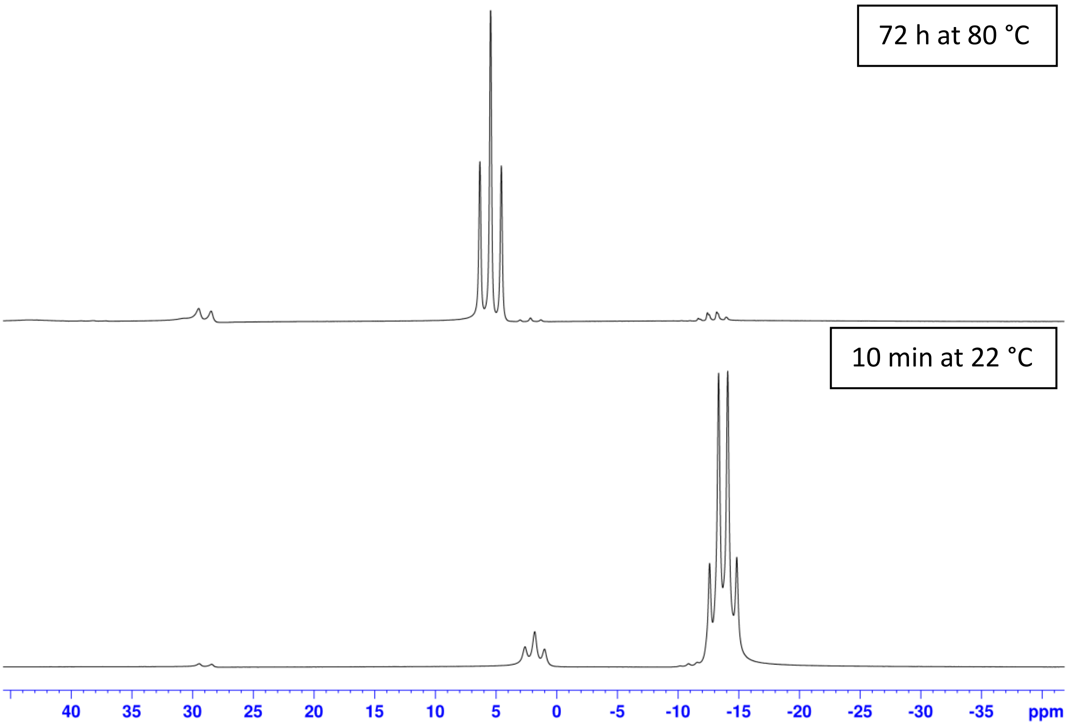

^{1}H NMR spectra

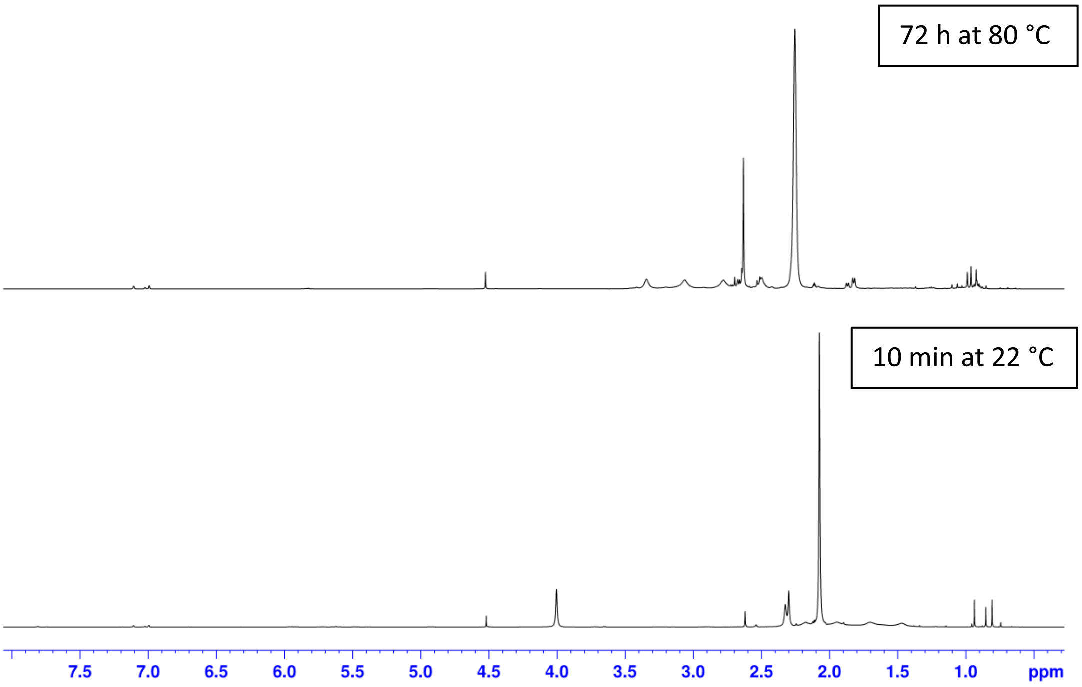

Figure S3: Catalytic dehydrocoupling of dimethylamine borane with **1t**K (2.5 mol%) in d_8-toluene over 144 h. at 80 °C.

^{11}B NMR spectra

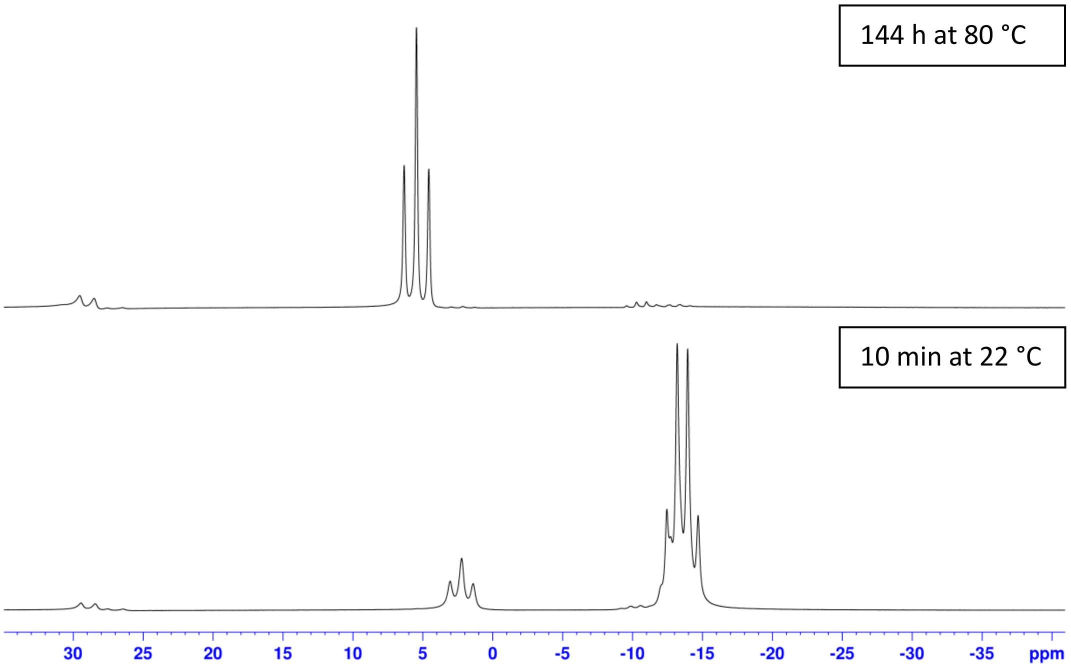

^{1}H NMR spectra

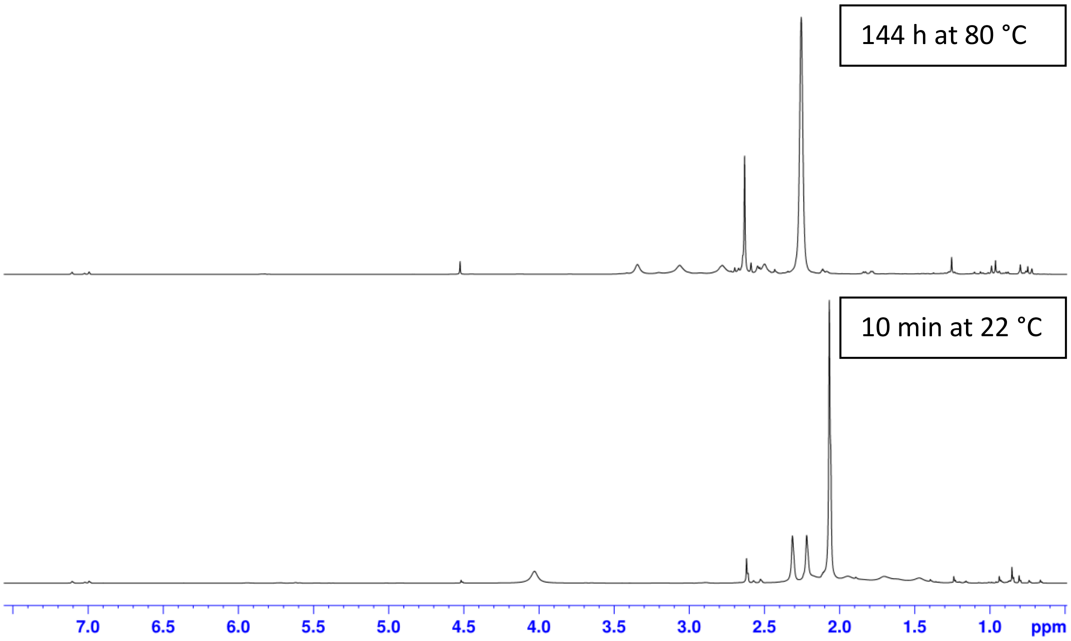

Figure S4: Catalytic dehydrocoupling of dimethylamine borane with **1t**Li (2.5 mol%) in d_{12}-cyclohexane over 168 h. at 80 °C.

^{11}B NMR spectra

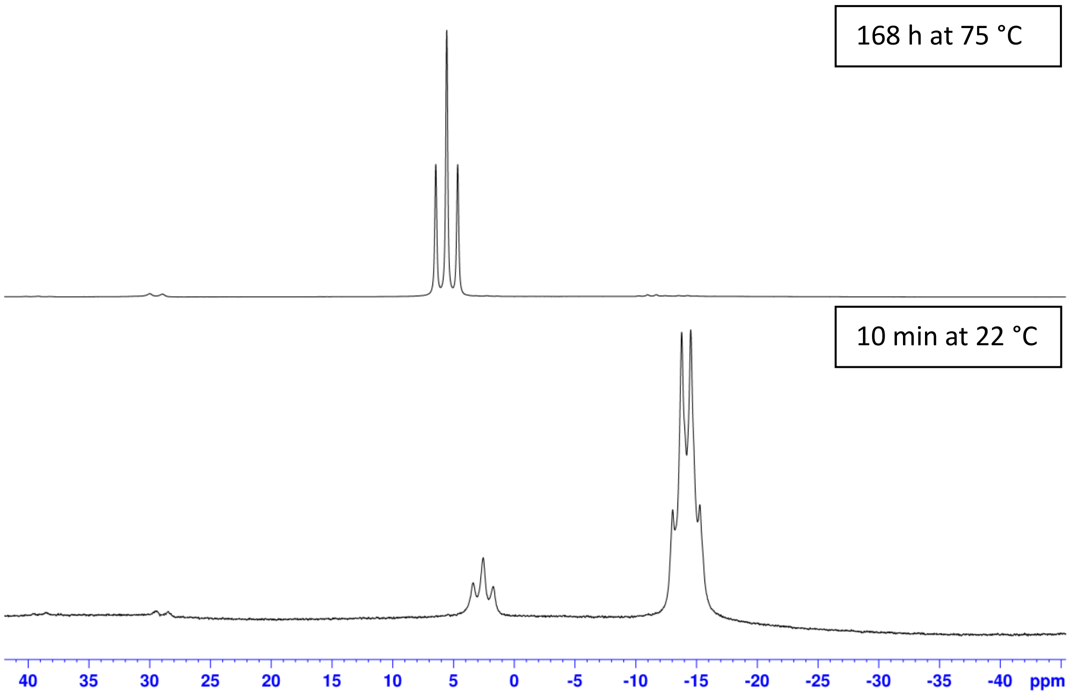

^{1}H NMR spectra

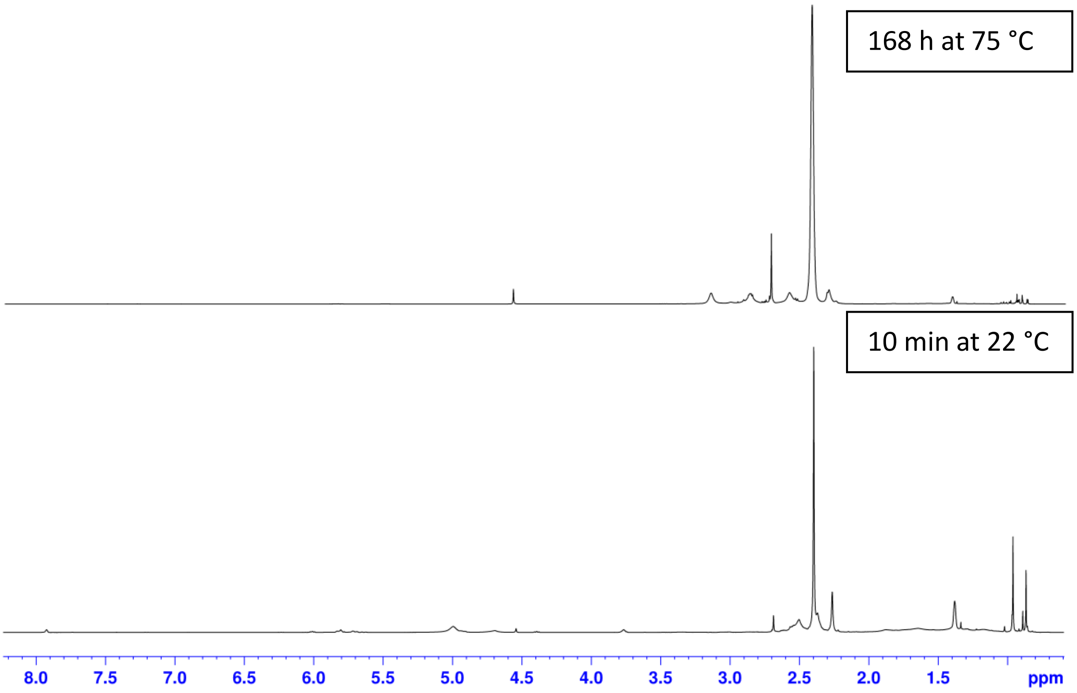

Figure S5: Catalytic dehydrocoupling of dimethylamine borane with **1t**Li (2.5 mol%) in d_8-thf over 360 h. at 65 °C.

^{11}B NMR spectra

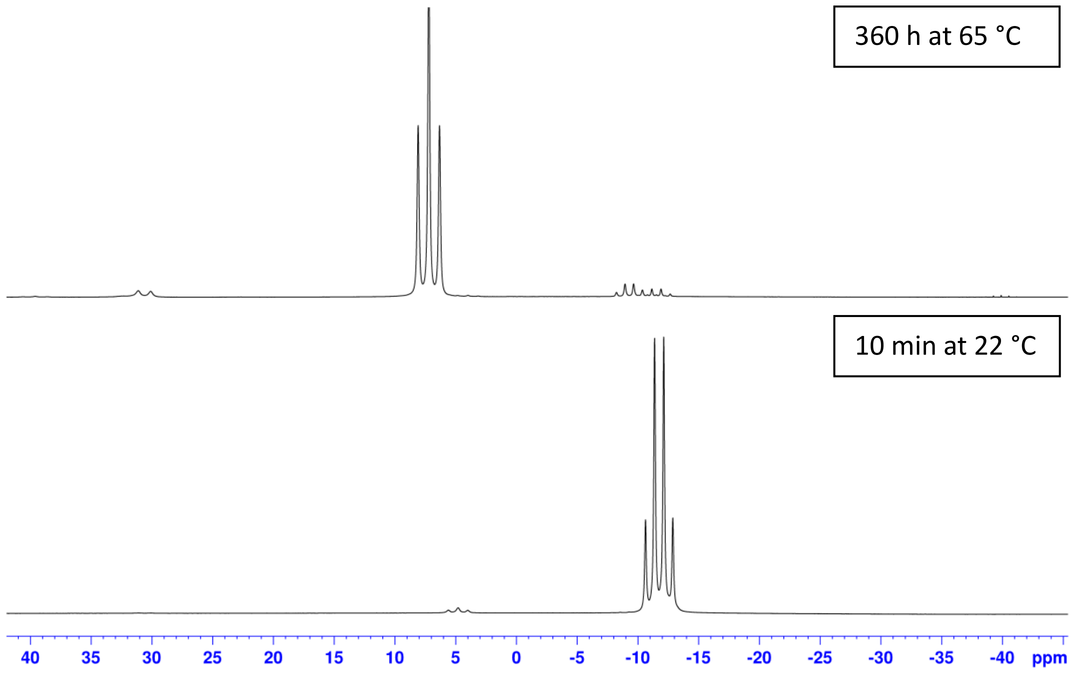

^{1}H NMR spectra

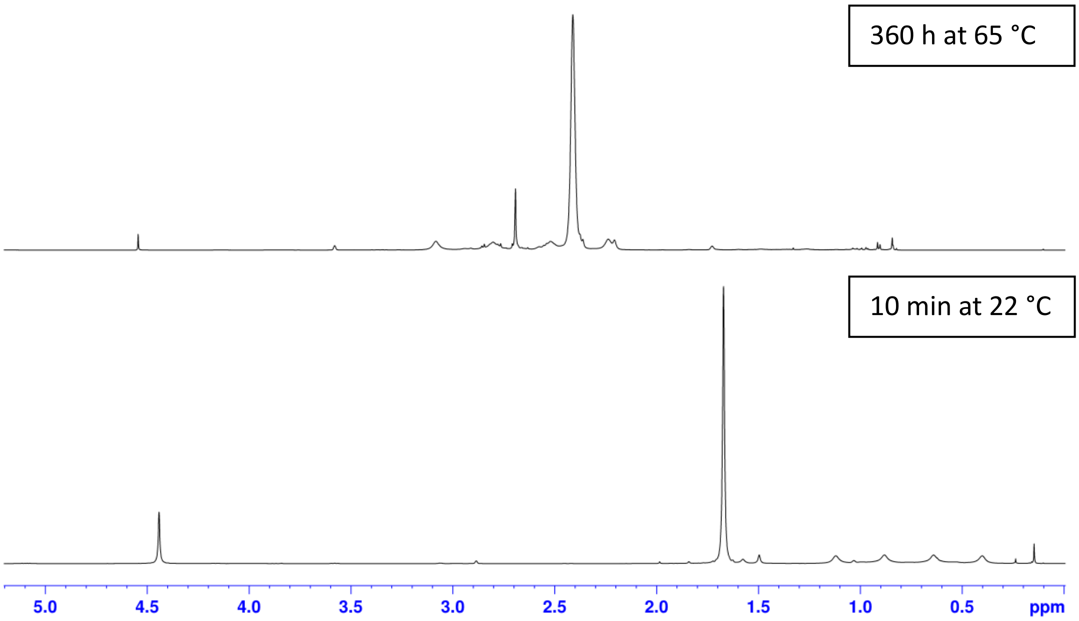

Figure S6: Catalytic dehydrocoupling of dimethylamine borane with **1t**Li·AEE (2.5 mol%) in d_8-toluene over 120 h. at 80 °C.

^{11}B NMR spectra

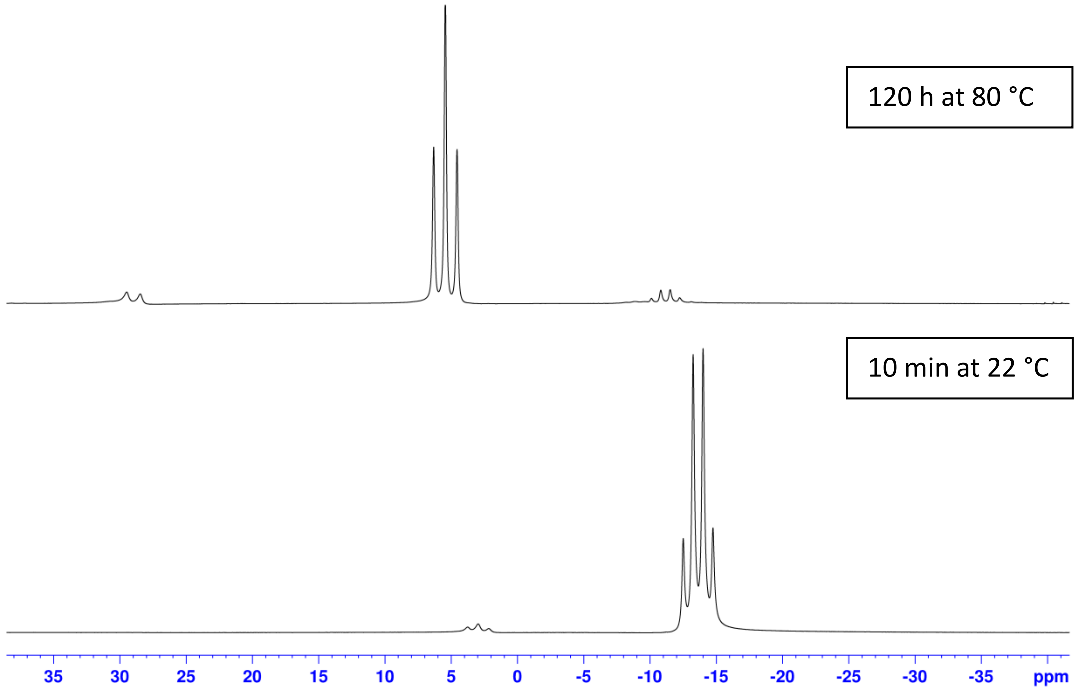

^{1}H NMR spectra

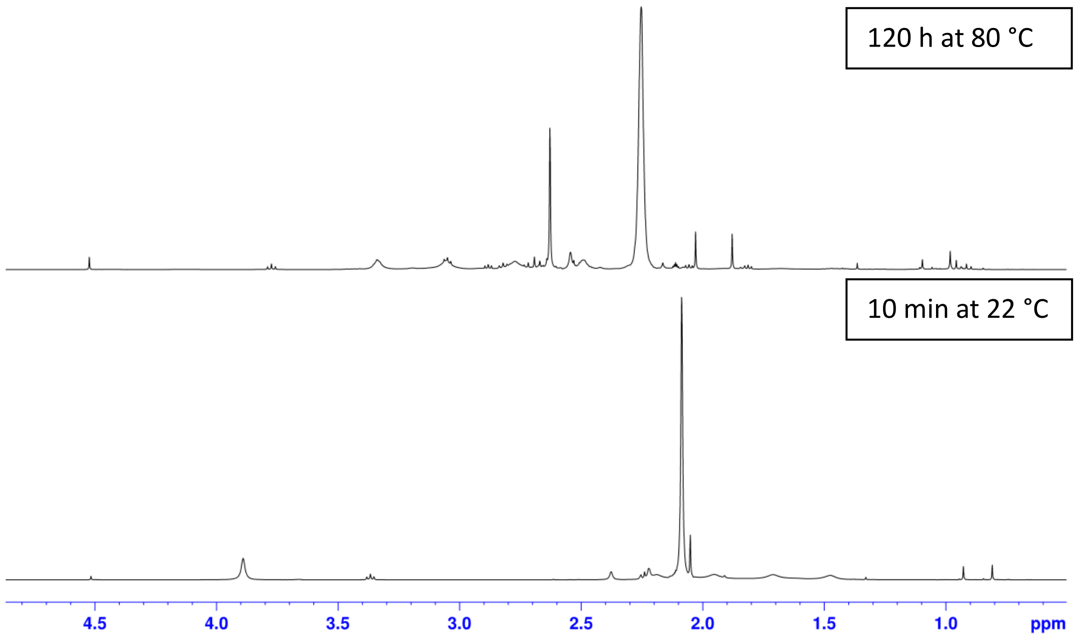

Figure S7: Catalytic dehydrocoupling of dimethylamine borane with **1t**Li (2.5 mol%) in d_5-pyridine over 5 h. at 80 °C.

^{11}B NMR spectra

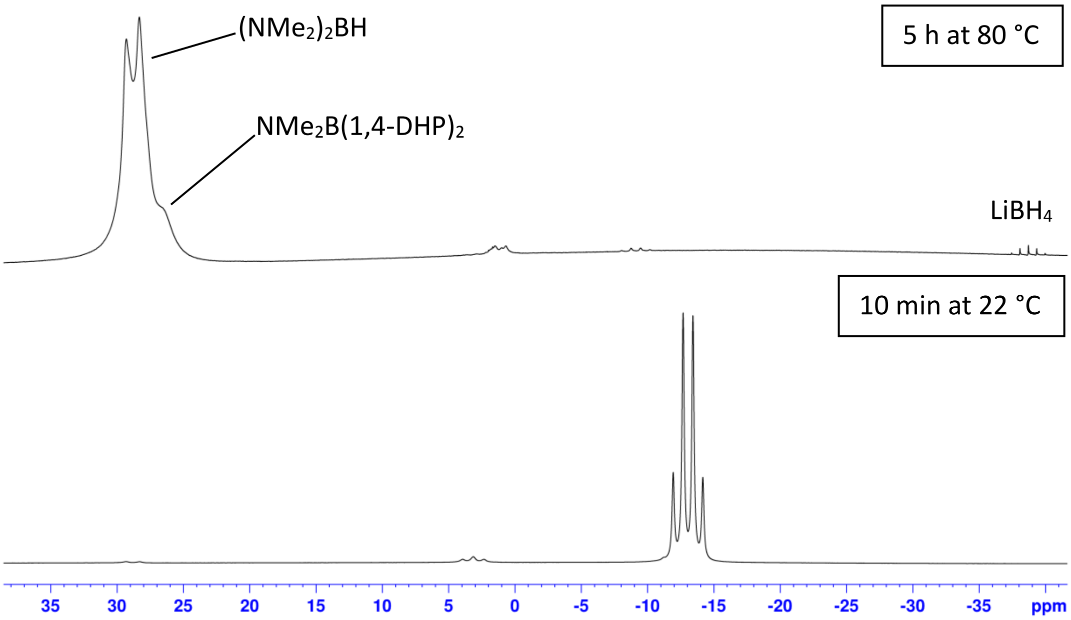

^{1}H NMR spectra

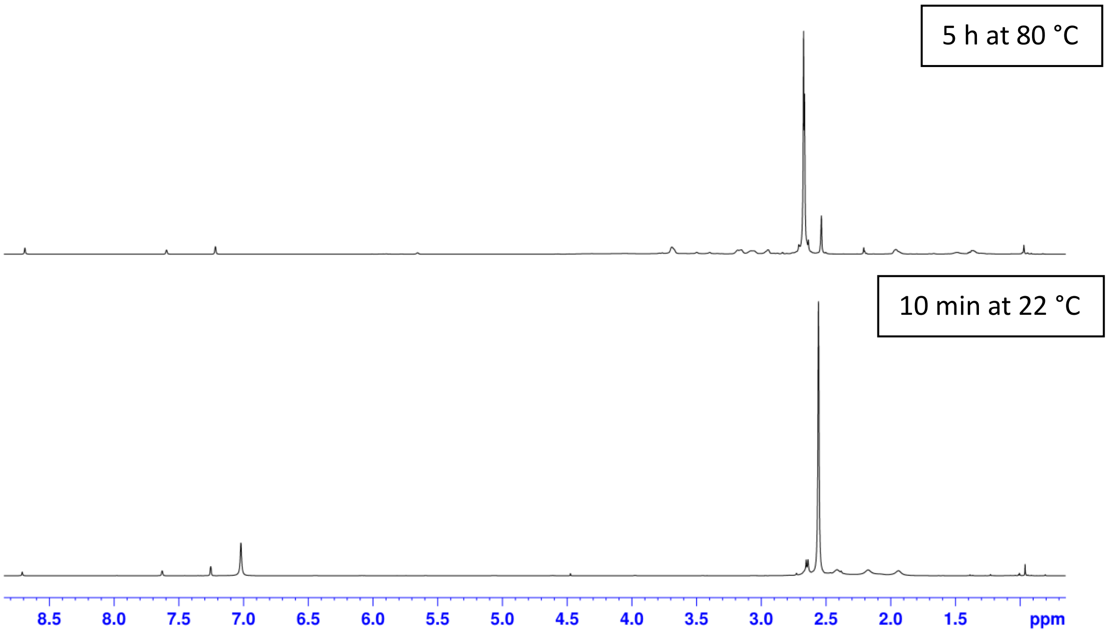

Figure S8: $^{11}B\{^1H\}$ spectra of reaction between $HNMe_2{\cdot}BH_3$ in *d*$_5$-pyridine at 80 ˚C for 20 h. Reaction shows approximately 85 % $HNMe_2{\cdot}BH_3$ and 15% pyrine$\cdot BH_3$ adduct.

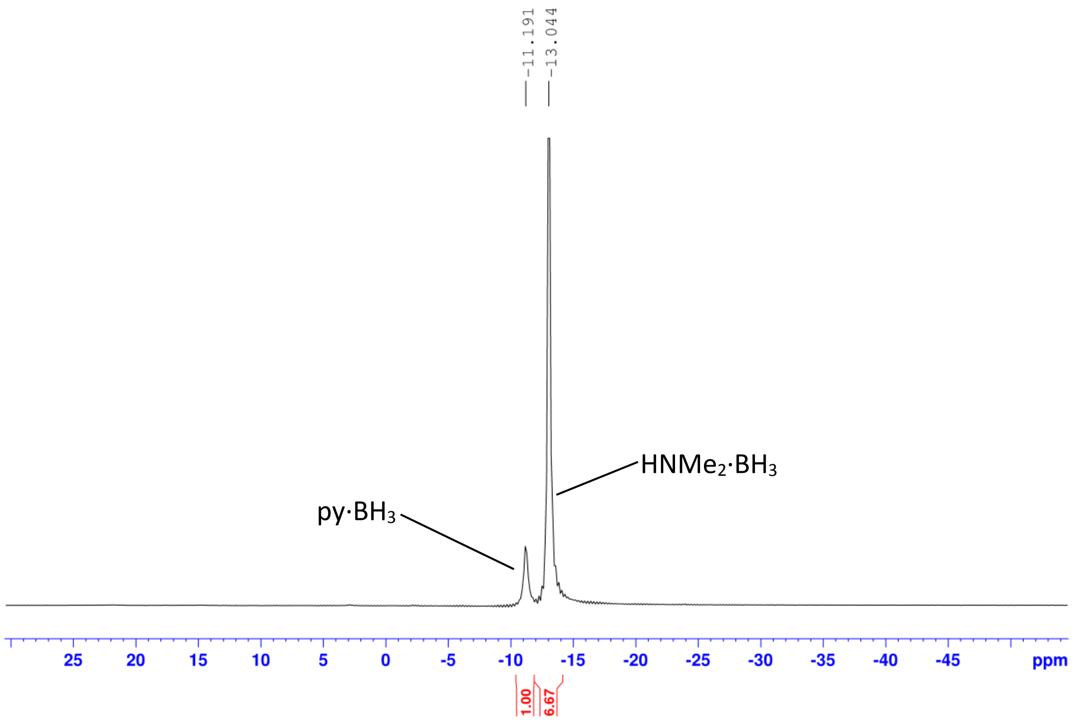

Figure S9: Catalytic dehydrocoupling of dimethylamine borane with **2** (1.25 mol%) in d_5-pyridine over 5 h. at 80 °C.

^{11}B NMR spectra

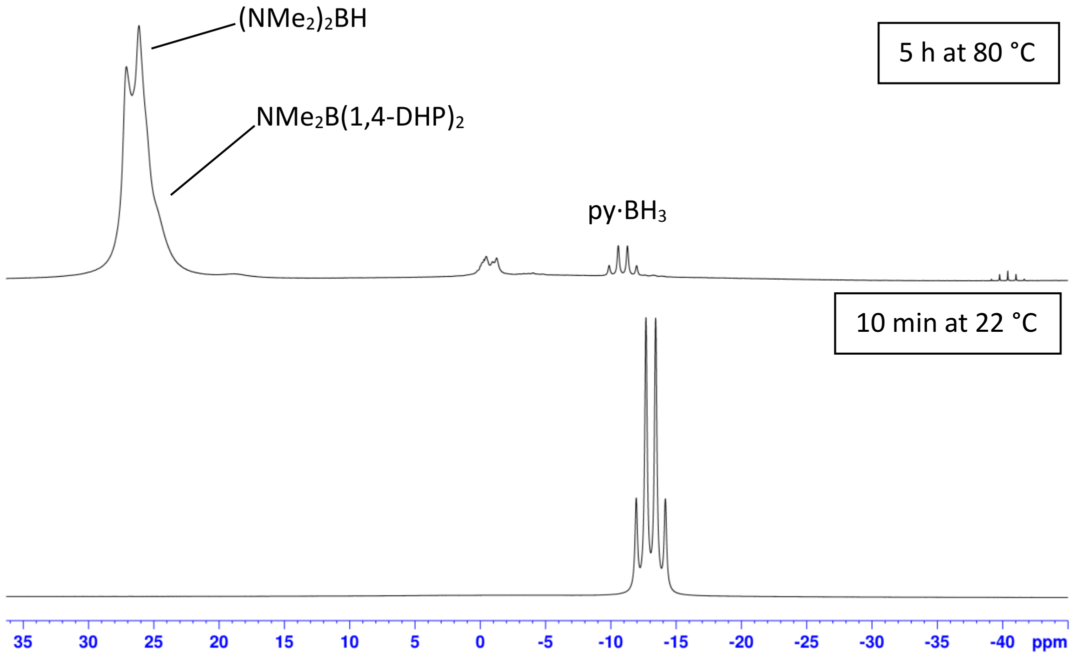

^{1}H NMR spectra

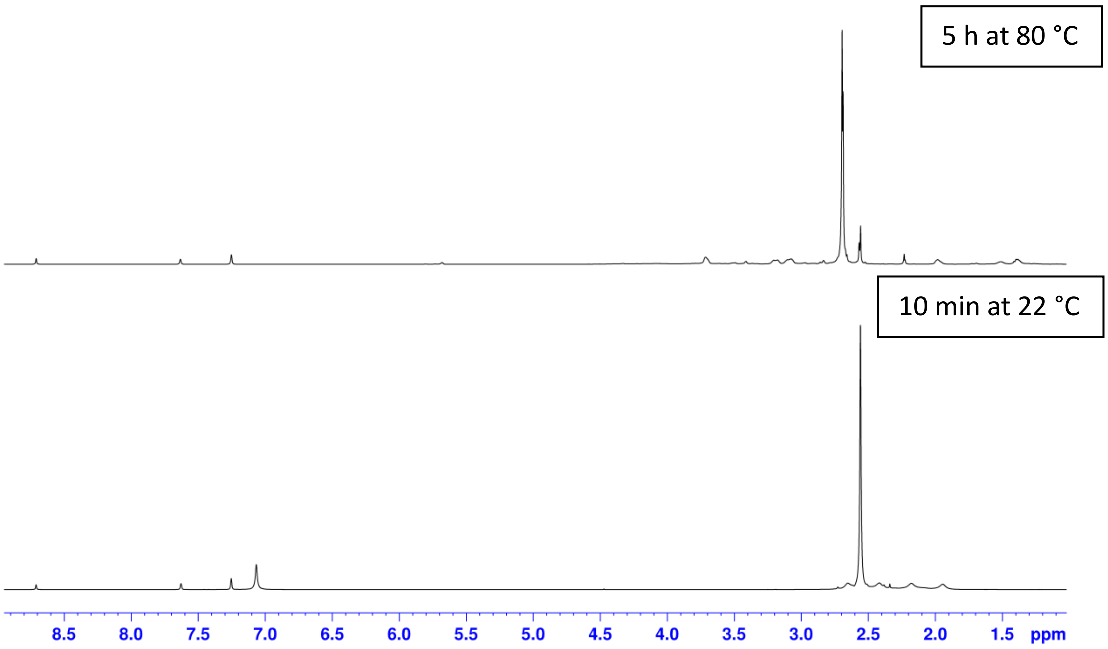

Figure S10: Catalytic dehydrocoupling of dimethylamine borane with **2** (1.25 mol%) in d_8-toluene over 146 h. at 80 °C.

^{11}B NMR spectra

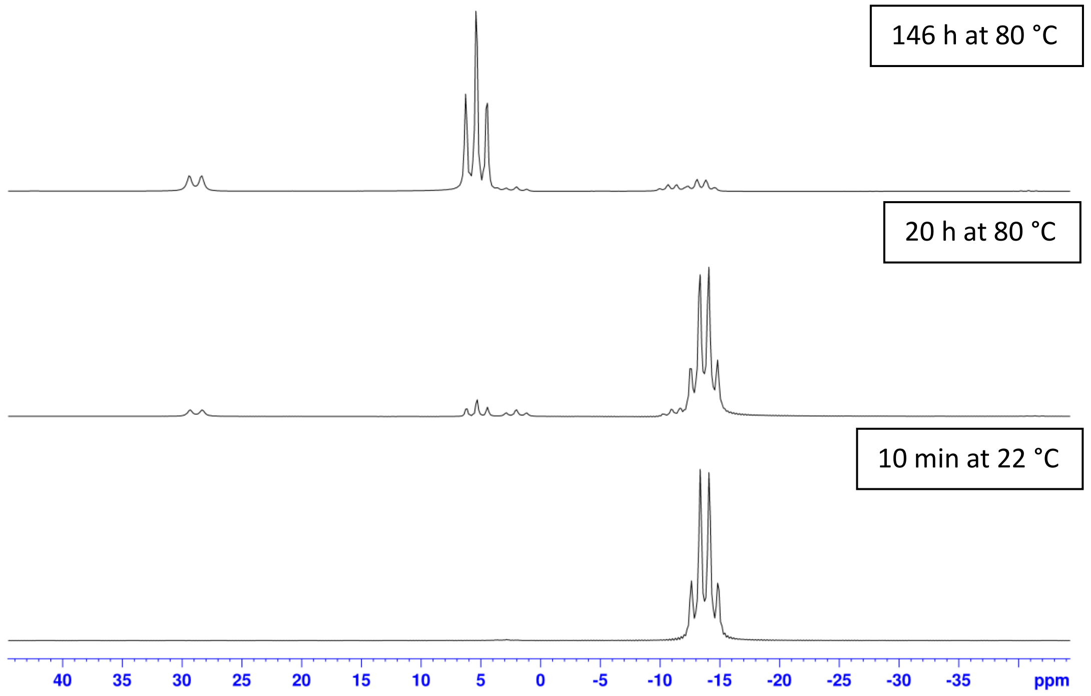

^{1}H NMR spectra

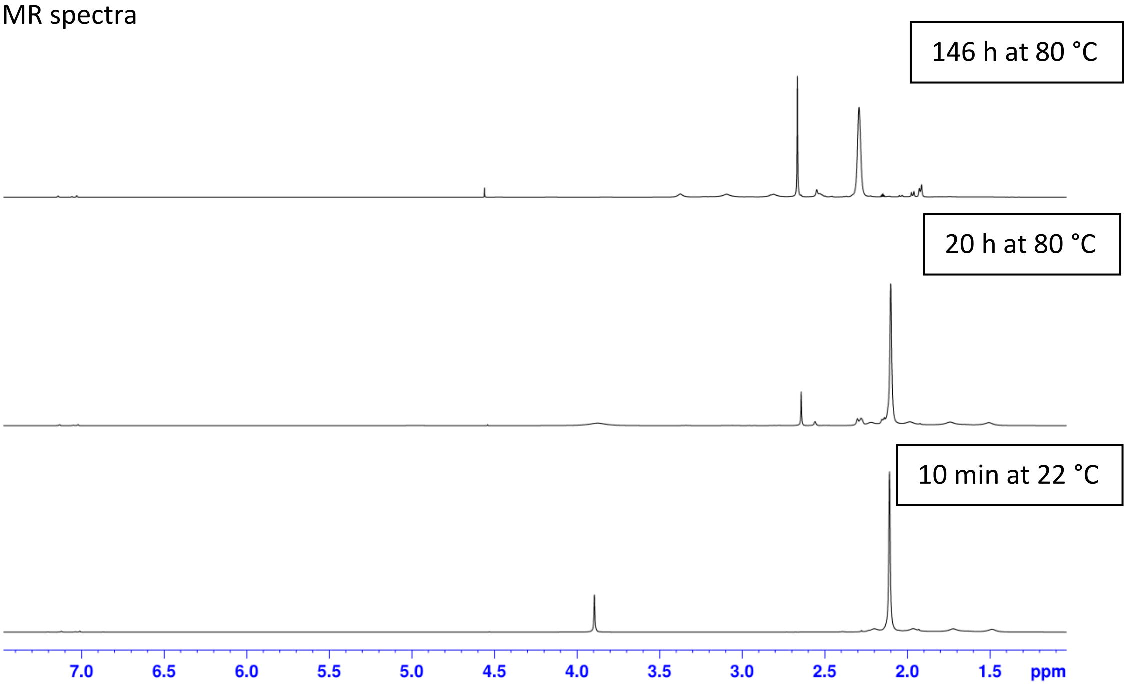

Figure S11: Catalytic dehydrocoupling of dimethylamine borane with $LiAlH_4$ in d_5-pyridine over 60 h. at 80 °C. In this experiment the resonance corresponding to $(NMe_2)_2BH$ is the main product after heating for 9 hours. Moreover, the starting material is fully consumed at this point. At this point the second product is minor but begins to increase with prolonged heating, indicating that the former (**III**) is transformed into the latter (**VI**).

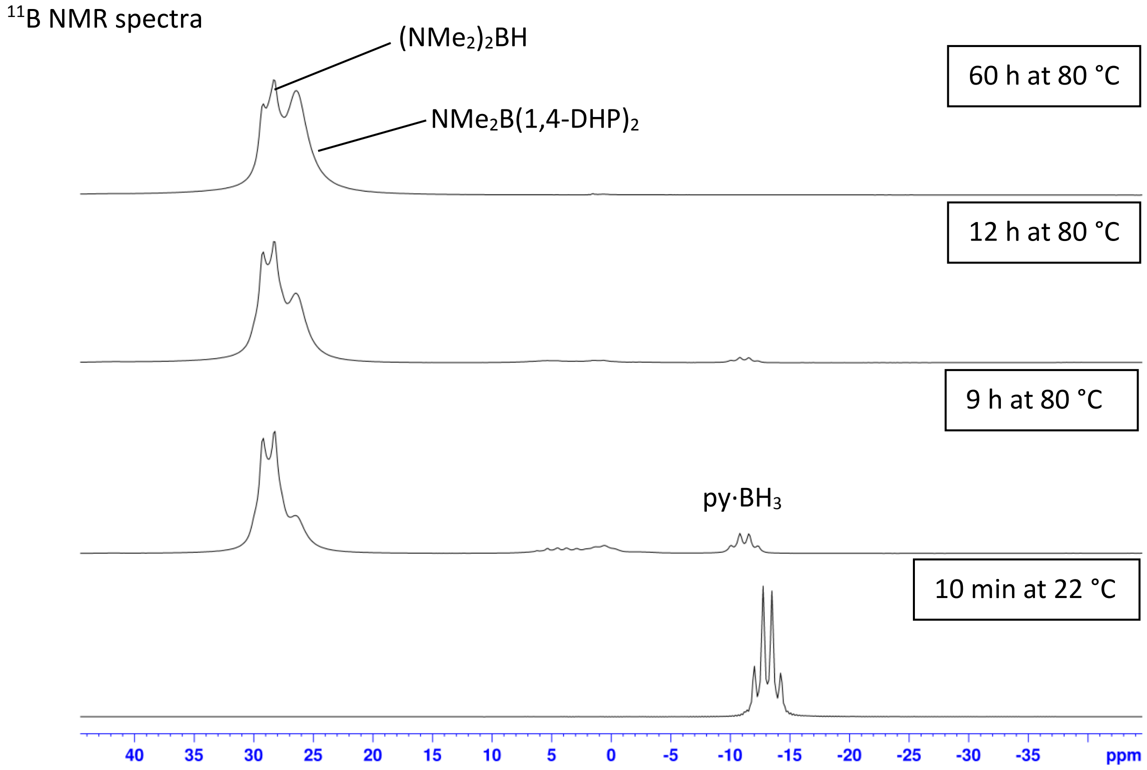

Synthesis of **VI**: Dimethylamine borane (118 mg, 2 mmol) and $LiAlH_4$ (76 mg, 2 mmol) were dissolved in pyridine (4 mL). The reaction was stirred at 80 °C for 18 h and then filtered through a celite pad. The celite was washed with three portions of THF (5 mL) and the filtrate was placed at -30 °C overnight. The resulting solid was washed with hexane and all volatiles subsequently removed from the filtrate affording **VI** as viscous white oil, 168 mg, 39% based upon dimethylamine borane.

Figure S12: Spectroscopic characterisation of **VI** in C_6D_6.

^{1}H NMR (C_6D_6): δ 5.96 (4H, dt, DHP-C*H*, $^3J_{H\text{-}H}$ 8.0 Hz, $^4J_{H\text{-}H}$ 1.7 Hz), 4.53 (4H, dt, DHP-C*H*, $^3J_{H\text{-}H}$ 8.6 Hz, $^3J_{H\text{-}H}$ 3.4 Hz), 2.96 (4H m, DHP-C*H₂*) and 2.31 ppm (6H, s, N*Me₂*).

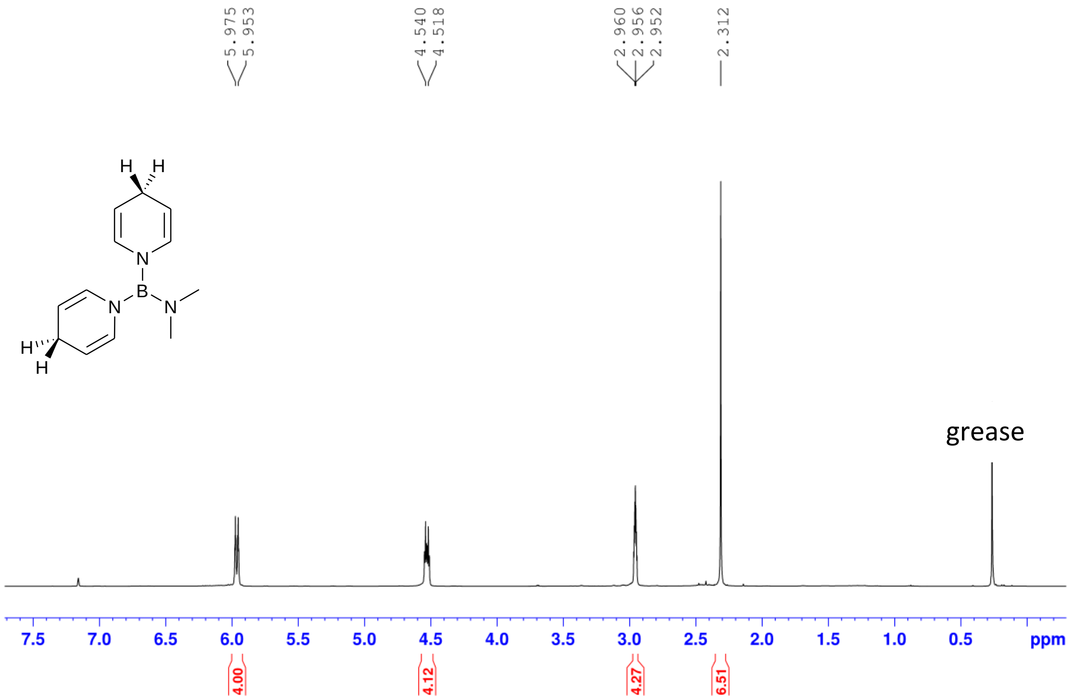

^{11}B NMR (C_6D_6): δ 26.4 ppm

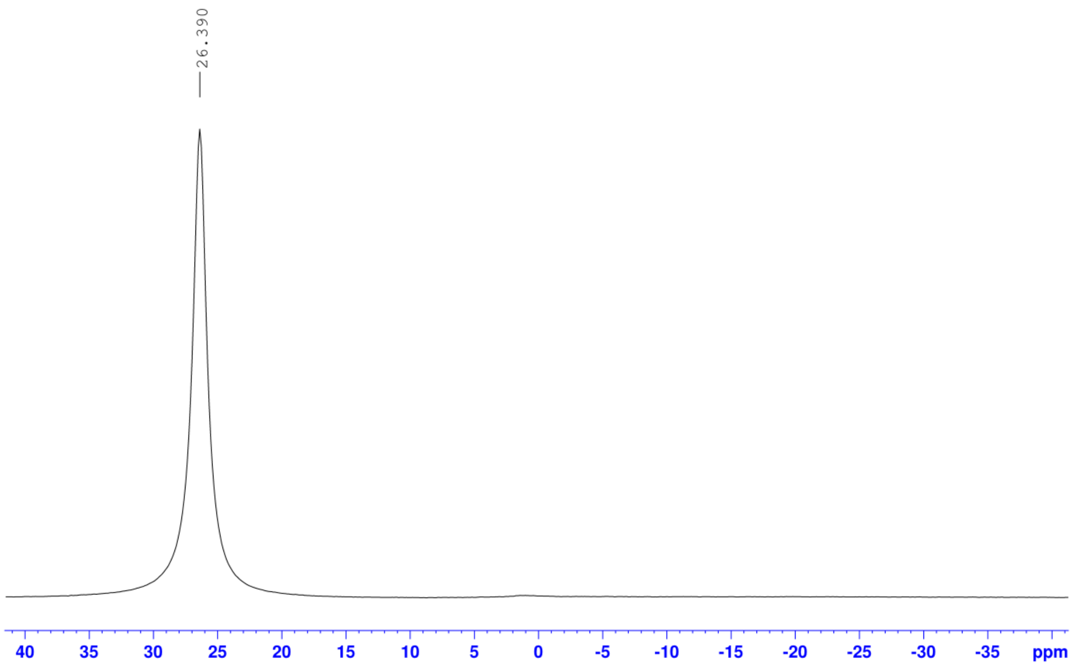

^{13}C NMR (C_6D_6): δ 130.2 (DHP –*C*H), 101.2 (DHP-*C*H), 39.1 (DHP-*C*H_2) and 23.3 ppm (N*Me₂*).

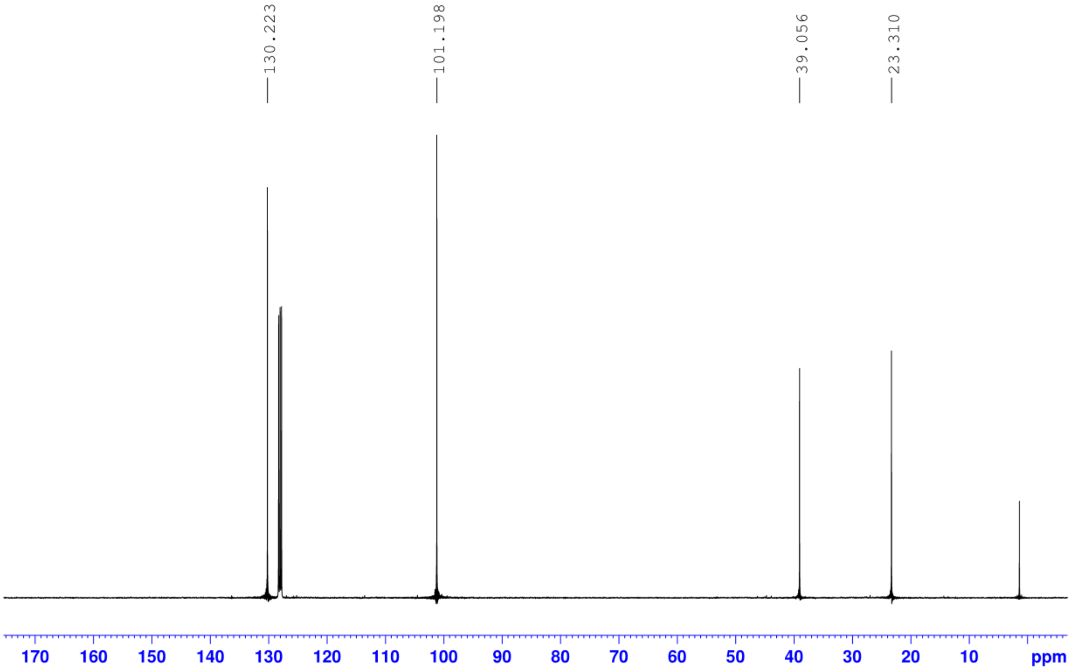

Figure S13: Catalytic dehydrocoupling of dimethylamine borane with **1t**Na (2.5 mol%) in d_5-pyridine over 8 h. at 80 °C.

^{11}B NMR spectra

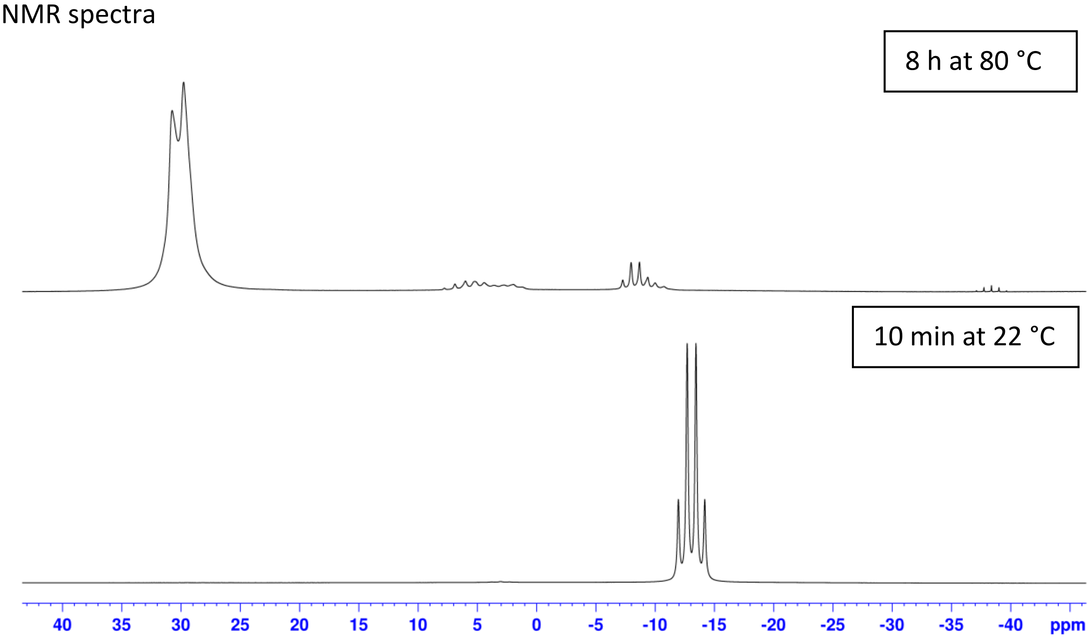

^{1}H NMR spectra

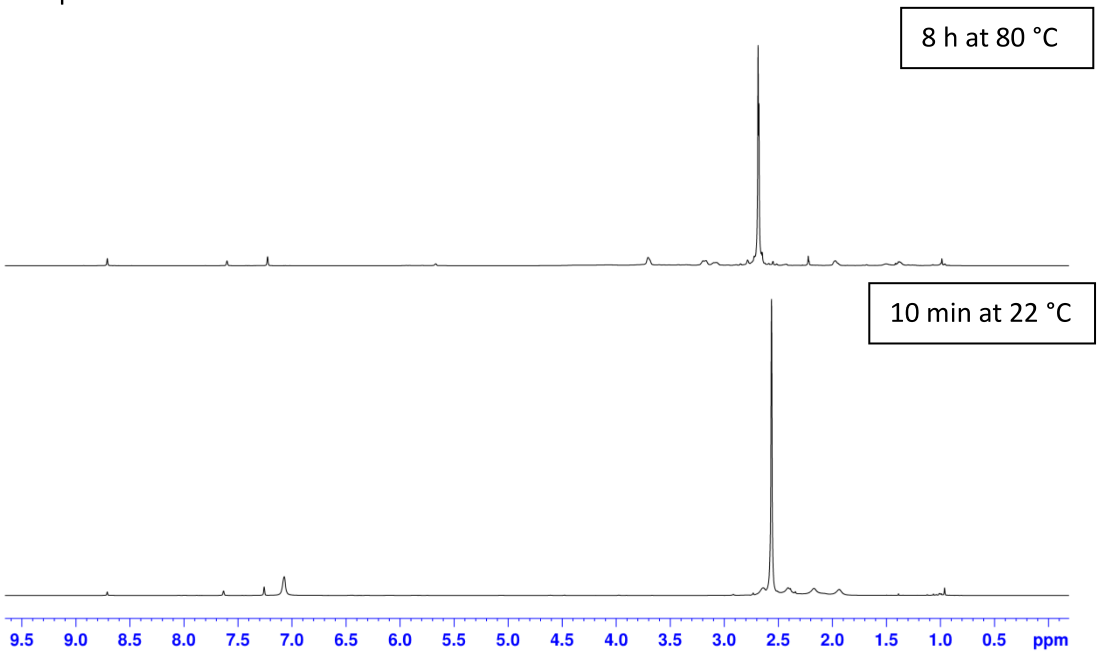

Figure S14: Catalytic dehydrocoupling of dimethylamine borane with **1t**K (2.5 mol%) in d_5-pyridine over 7 h. at 80 °C.

^{11}B NMR spectra

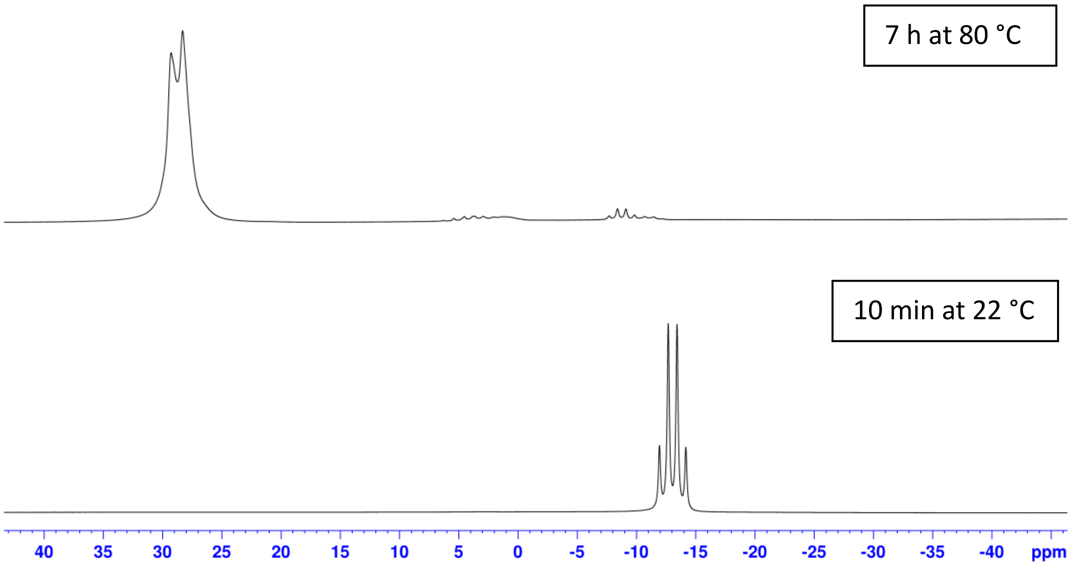

^{1}H NMR spectra

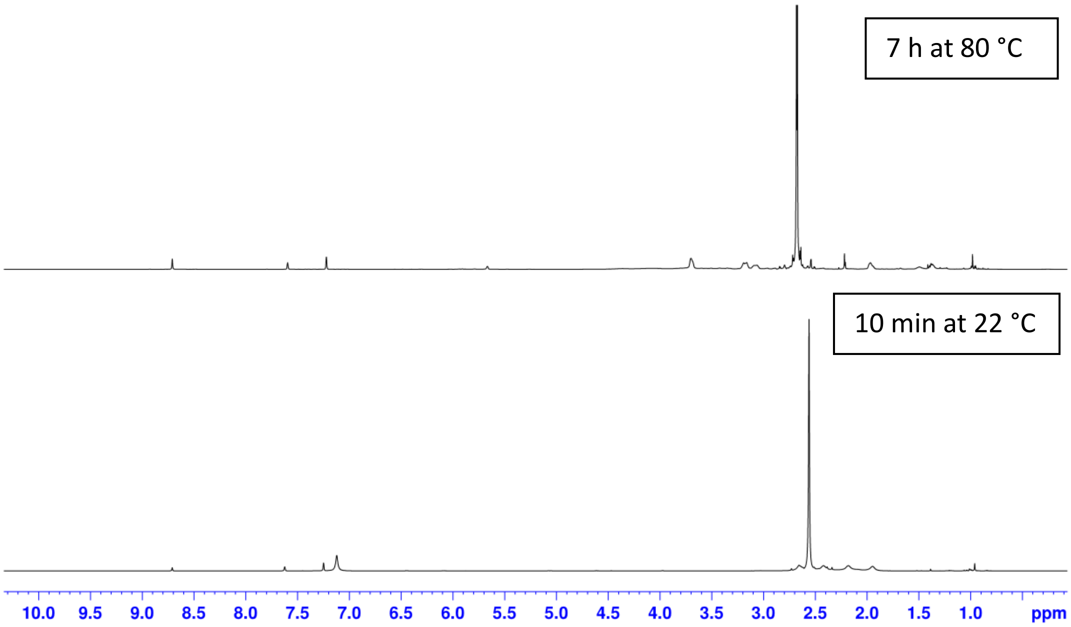

Figure S15: Catalytic hydroboration of benzaldehyde with HBPin using **1t**Li (5 mol%) in C_6D_6

^{1}H NMR spectra

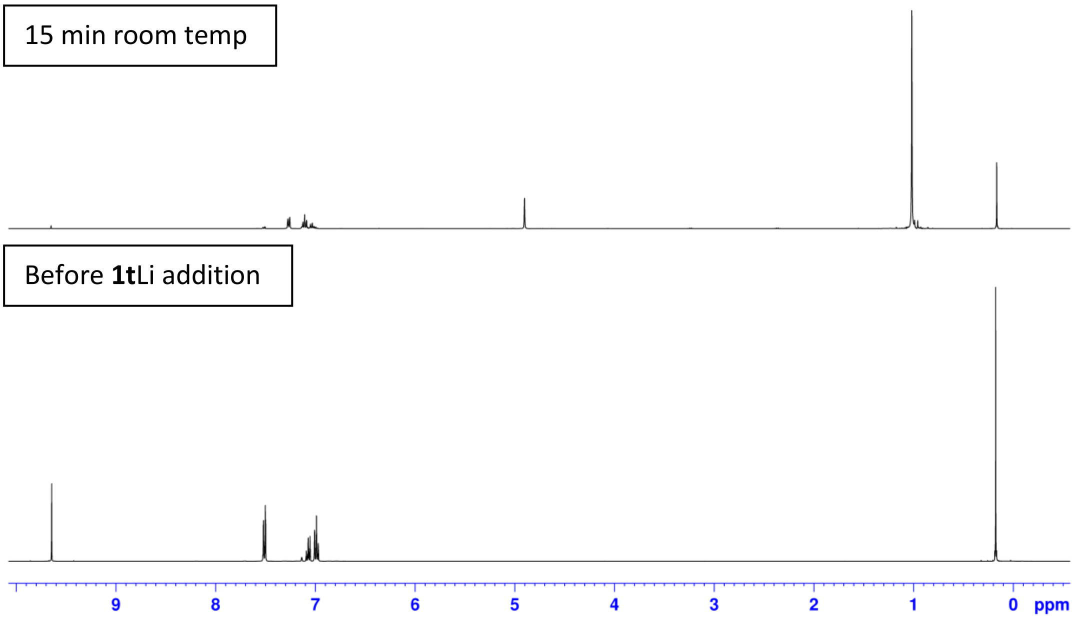

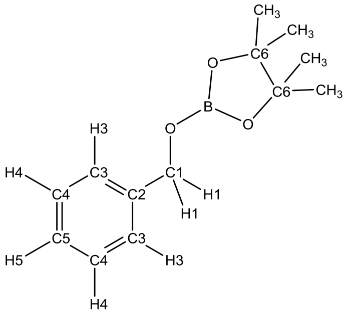

^{1}H NMR (400.1 MHz, C_6D_6, 300K): δ 7.30 (2H, d, H3), 7.17-7.09 (2H, m, H4), 7.08-7.02 (1H, m, H5), 4.95 (2H, s, H1) and 1.04 ppm (12H, s, CH_3 of BPin).

^{11}B NMR (128.38 MHz, C_6D_6, 300K): δ 22.8 ppm (s, O*B*Pin).

^{13}C NMR (100.62 MHz, C_6D_6, 300K): δ 140.0 (C2), 128.6 (C4), 127.6 (C5), 127.1 (C3), 82.7 (C6), 67.0 (C1) and 24.7 ppm (CH_3).

Figure S16: Catalytic hydroboration of 2-methoxybenzaldehyde with HBPin using **1t**Li (5 mol%) in C_6D_6

^{1}H NMR spectra

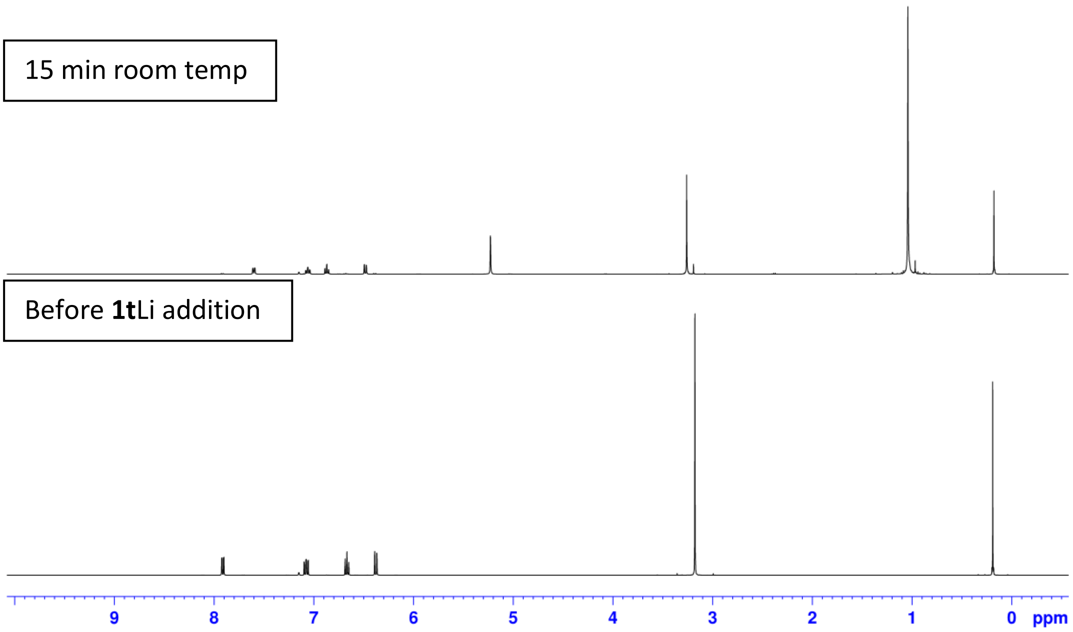

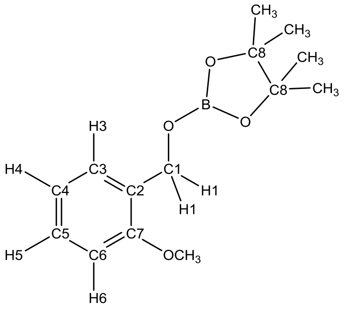

^{1}H NMR (400.1 MHz, C_6D_6, 300K): δ 7.66 (1H, d, $^3J_{H-H}$ = 7.29 Hz, *H*3), 7.07 (1H, t, $^3J_{H-H}$ = 8.12 Hz, *H*5), 6.89 (1H, t, $^3J_{H-H}$ = 7.46 Hz, *H*4), 6.48 (1H, t, $^3J_{H-H}$ = 8.18 Hz, *H*6), 5.29 (2H, s, H1), 3.24 (3H, s, OCH_3) and 1.05 ppm (12H, s, CH_3 of BPin).

^{11}B NMR (128.38 MHz, C_6D_6, 300K): 22.9 ppm (s, O*B*Pin).

^{13}C NMR (100.62 MHz, C_6D_6, 300K): δ 156.8 (C7), 128.4 (C5), 127.5 (C3), 120.8 (C4), 110.1 (C6), 82.7 (C8), 62.6 (C1), 54.7 (OCH_3) and 24.7 ppm (CH_3 of BPin).

Figure S17: Catalytic hydroboration of 2-napthaldehyde with HBPin using **1t**Li (5 mol%) in C_6D_6

^{1}H NMR spectra

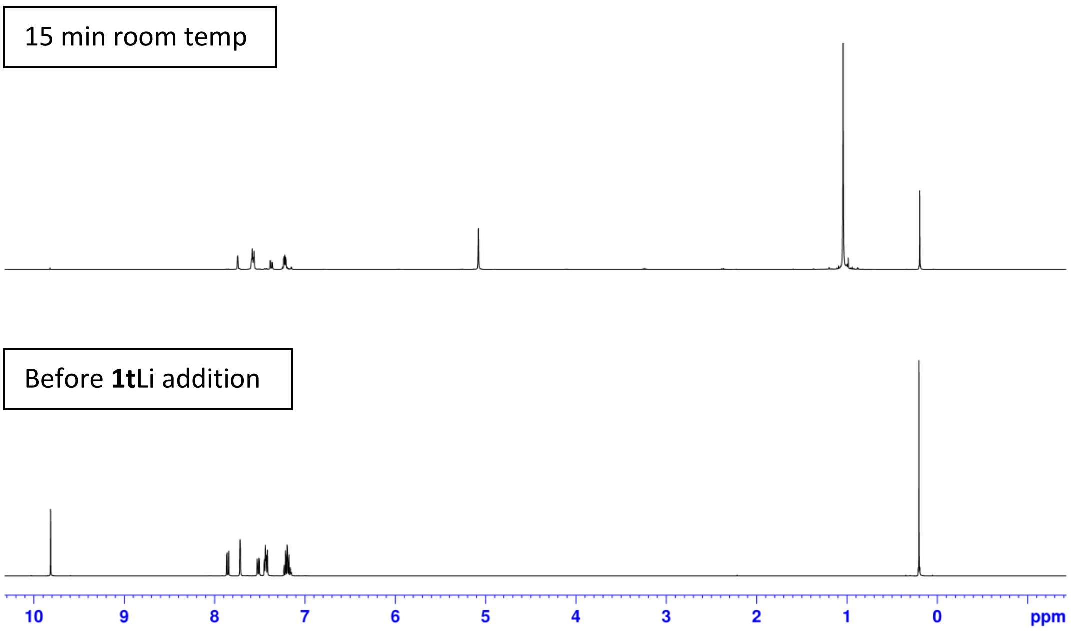

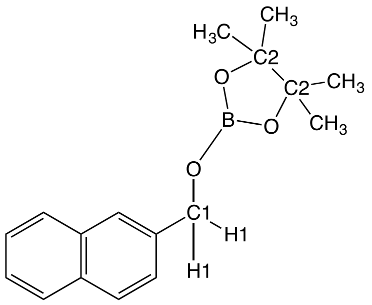

^{1}H NMR (400.1 MHz, C_6D_6, 300K): δ 7.77 (1H, s, Ar-*H*), 7.62-7.56 (3H, m, Ar-*H*), 7.39 (1H, d, Ar-*H*), 7.25-7.21 (2H, m, Ar-*H*), 5.10 (2H, s, H1) and 1.05 ppm (12H, s, CH_3 of BPin).

^{11}B NMR (128.38 MHz, C_6D_6, 300K): δ 22.9 ppm (s, O*B*Pin).

^{13}C NMR (100.62 MHz, C_6D_6, 300K): δ 137.5 (quat Ar-*C*), 134.0 (quat Ar-*C*), 133.4 (quat Ar-*C*), 128.4 (Ar-*C*), 128.3 (Ar-*C*), 128.0 (Ar-*C*), 126.2 (Ar-*C*), 125.9 (Ar-*C*), 125.7 (Ar-*C*), 125.3 (Ar-*C*), 82.8 (C2), 67.1 (C1) and 24.7 ppm (CH_3 of BPin).

Figure S18: Catalytic hydroboration of ferrocene carboxaldehyde with HBPin using **1t**Li (5 mol%) in C_6D_6

^1H NMR spectra

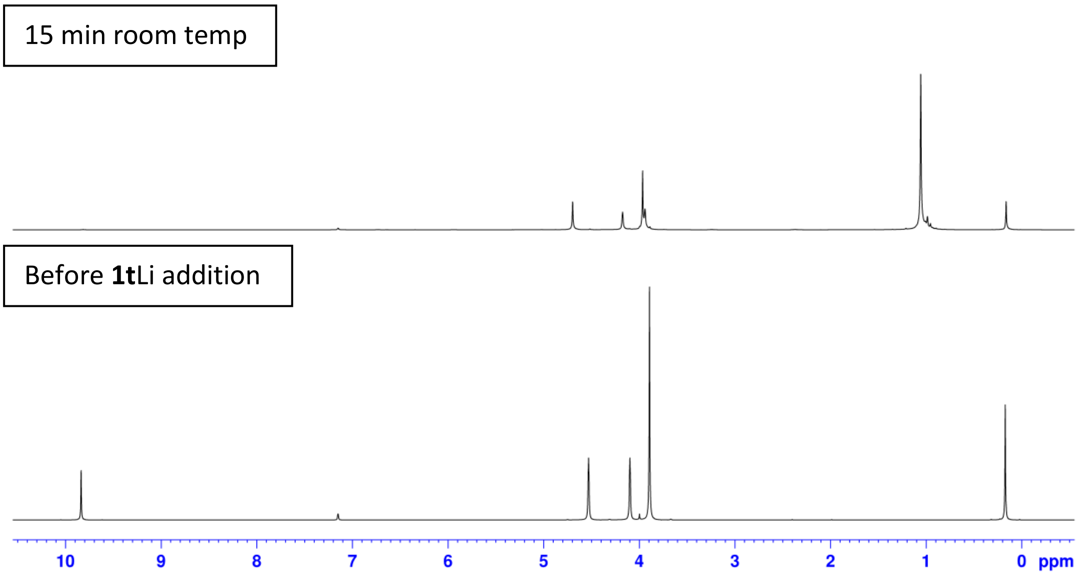

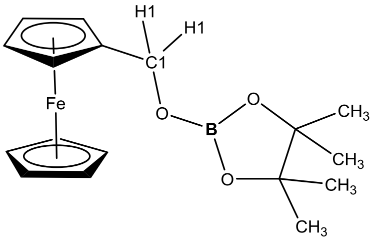

^{1}**H NMR** (400.1 MHz, C_6D_6, 300K): δ 4.74 (2H, s, H1), 4.20 (2H, t, H), 3.98 (5H, s, Cp ring), 3.95 (2H, t, H) and 1.07 ppm (12H, s, CH_3 of BPin).

^{11}**B NMR** (128.38 MHz, C_6D_6, 300K): δ 22.6 ppm (O*B*Pin).

^{13}**C NMR** (100.62 MHz, C_6D_6, 300K): δ 86.1 (C2), 82.6 (C6), 69.0 (C4), 68.8 (C5), 68.5 (C3), 63.4 (C1) and 24.8 ppm (CH_3 of BPin).

Figure S19: Catalytic hydroboration of 4-bromobenzaldehyde with HBPin using **1t**Li (5 mol%) in C_6D_6

^{1}H NMR spectra

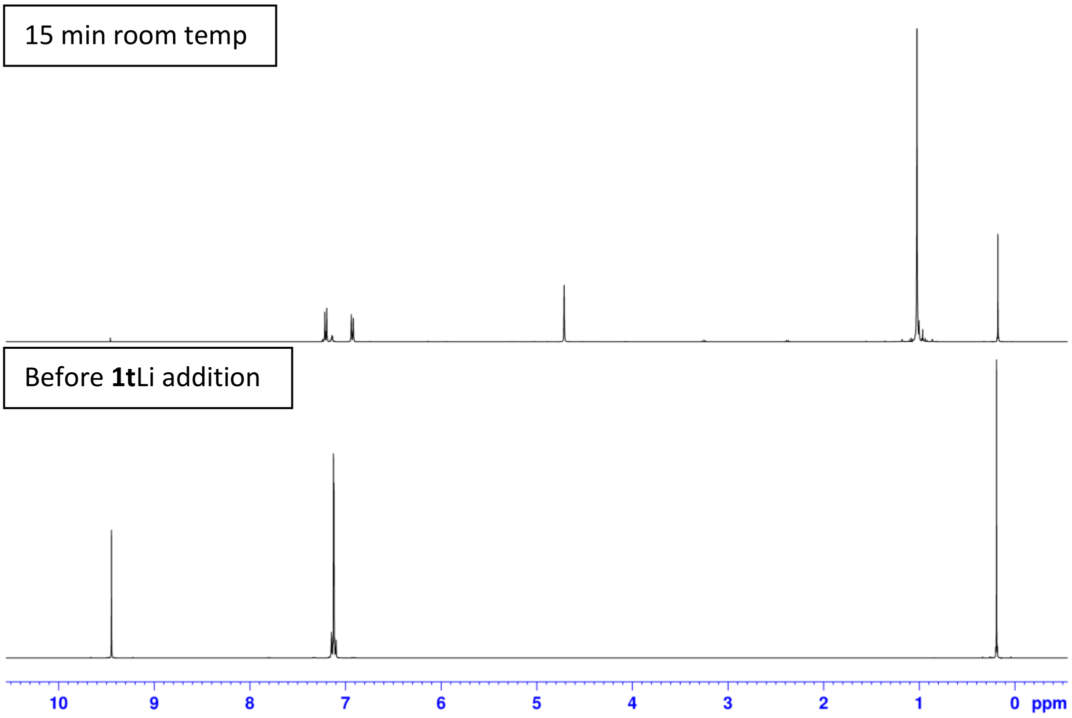

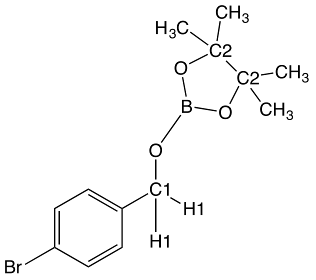

^{1}H NMR (400.13 MHz, C_6D_6, 300K): δ 7.22 (2H, d, $^3J_{H\text{-}H}$ = 8.31 Hz, Ar-H), 6.93 (2H, d, $^3J_{H\text{-}H}$ = 8.31 Hz, Ar-H), 4.73 (2H, s, H1, C*1*) and 1.03 ppm (12H, s, CH_3 of BPin).

^{11}B NMR (128.38 MHz, C_6D_6, 300K): δ 22.8 ppm (s, *B*Pin).

^{13}C NMR (100.62 MHz, C_6D_6, 300K): δ 138.9 (quat Ar-*C*), 131.7 (Ar-*C*), 128.7 (Ar-*C*), 121.5 (quat Ar-*C*), 82.9 (C2), 66.1 (C1) and 24.7 ppm (CH_3 of BPin).

Figure S20: Catalytic hydroboration of mesitaldehyde with HBPin using **1t**Li (5 mol%) in C_6D_6

^{1}H NMR spectra

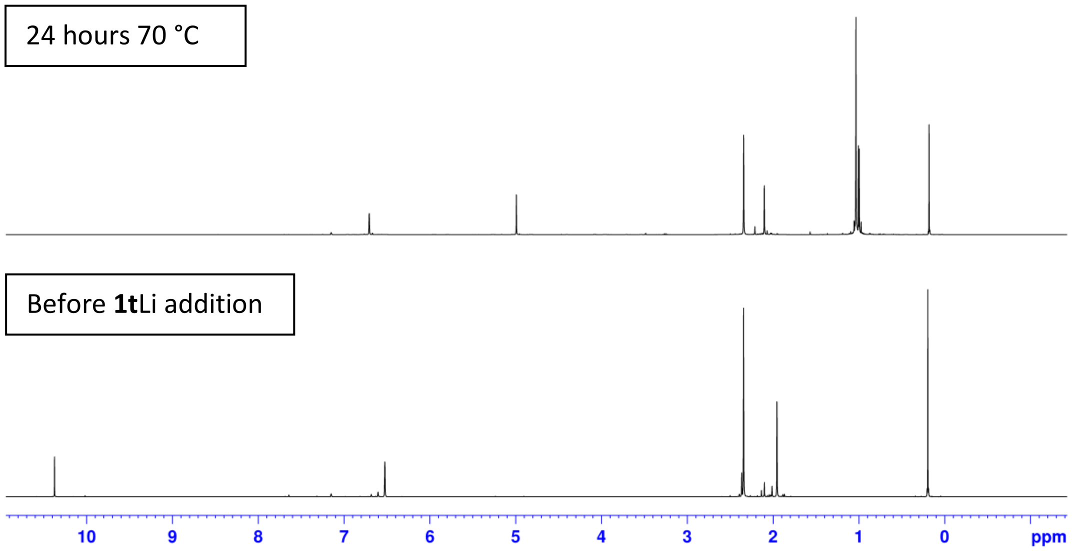

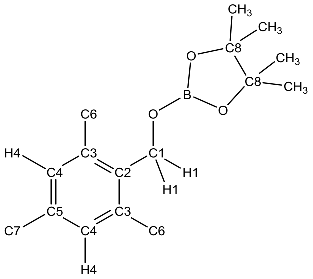

^{1}H NMR (400.1 MHz, C_6D_6, 300K): δ 6.71 (2H, br s, H4), 4.99 (2H, s, H1), 2.34 (6H, s, *o*-CH_3), 2.10 (3H, s, *p*-CH_3) and 1.03 ppm (12H, s, CH_3 of BPin).

^{11}B NMR (128.38 MHz, C_6D_6, 300K): δ 22.6 ppm (OBPin).

^{13}C NMR (100.62 MHz, C_6D_6, 300K): δ 136.9 (quat Ar-C), 136.5 (quat Ar-C), 132.1 (quat Ar-C), 128.4 (Ar-C), 81.6 (C8), 60.6 (C1), 23.8 (CH_3 of BPin), 20.1 (CH_3) and 18.7 ppm (CH_3).

Figure S21: Catalytic hydroboration of benzophenone with HBPin using **1t**Li (5 mol%) in C_6D_6

^{1}H NMR spectra

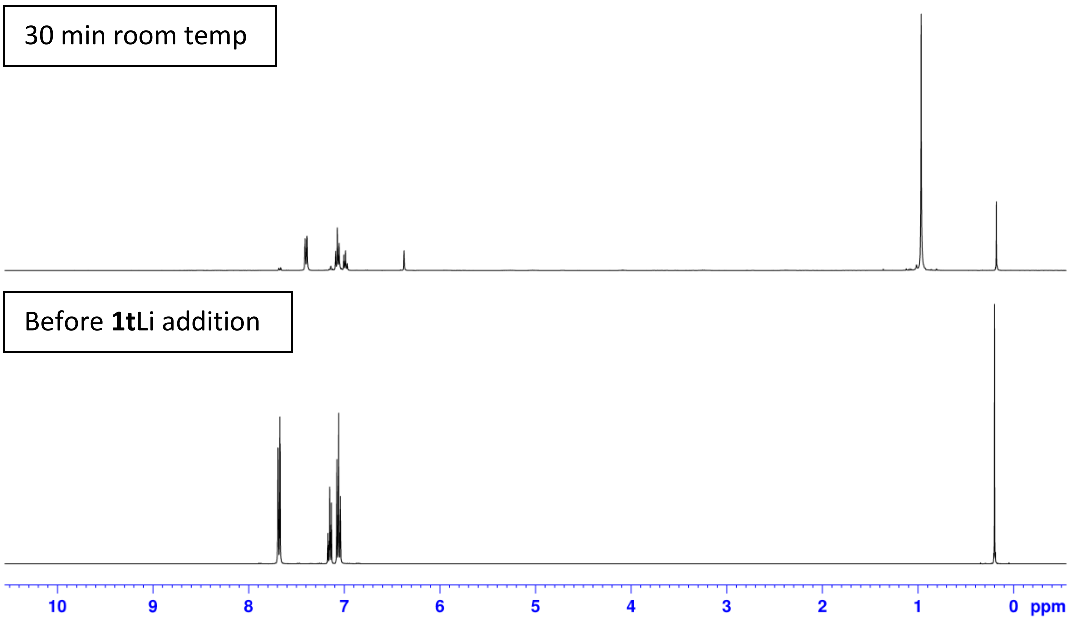

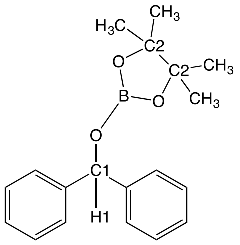

^{1}H NMR (400.1 MHz, C_6D_6, 300K): δ 7.45 (4H, d, Ar-H), 7.09 (4H, t, Ar-H), 7.00 (2H, t, Ar-H), 6.43 (1H, s, H1) and 0.98 ppm (12H, s, CH_3 of BPin).

^{11}B NMR (128.38 MHz, C_6D_6, 300K): δ 22.9 ppm (O*B*Pin).

^{13}C NMR (100.62 MHz, C_6D_6, 300K): δ 143.9 (quat Ar-C), 128.6 (Ar-C), 127.5 (Ar-C), 127.0 (Ar-C), 82.8 (C2), 78.6 (C1) and 24.6 ppm (CH_3 of BPin).

Figure S22: Catalytic hydroboration of 4-iodoacetophenone with HBPin using **1t**Li (5 mol%) in C_6D_6

^{1}H NMR spectra

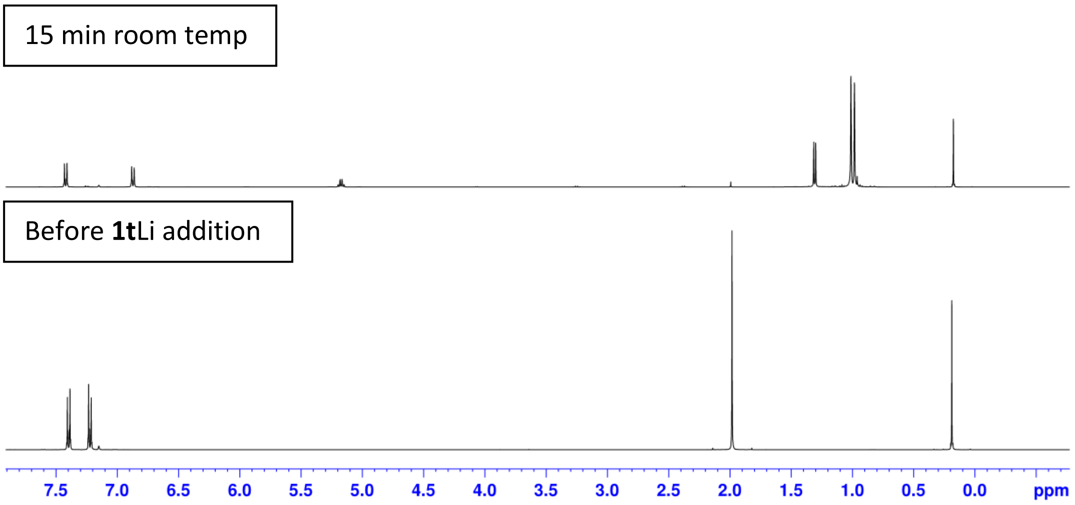

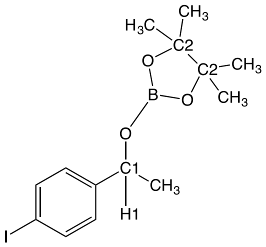

^{1}H NMR (400.1 MHz, C_6D_6, 300K): δ 7.43 (2H, d, Ar-H), 6.87 (2H, d, Ar-H), 5.21 (1H, q, H1), 1.32 (3H, d, CH_3) and 1.00 ppm (12H, d, CH_3 of BPin).

^{11}B NMR (128.38 MHz, C_6D_6, 300K): δ 22.5 ppm (O*B*Pin).

^{13}C NMR (100.62 MHz, C_6D_6, 300K): δ 137.7 (Ar-C), 127.7 (Ar-C), 92.7 (quat Ar-C), 82.7 (C2), 72.3 (C1), 25.6 (CH_3) and 24.7 ppm (CH_3 of Bpin).

Figure S23: Catalytic hydroboration of 2,2,2-trifluoroacetophenone with HBPin using **1t**Li (5 mol%) in C_6D_6

^{1}H NMR spectra

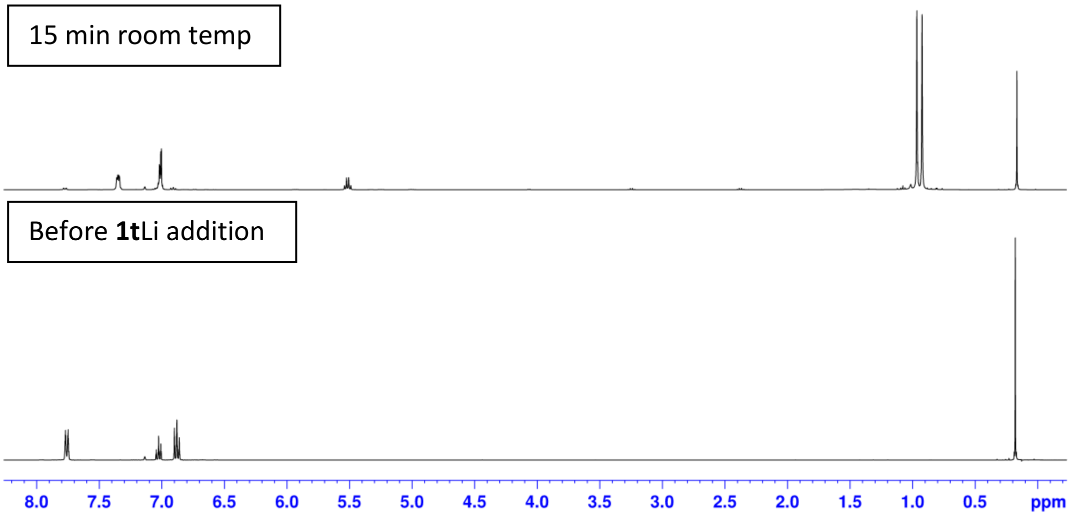

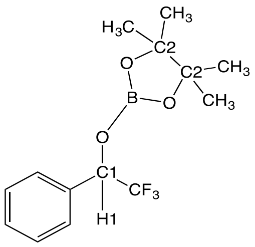

^{1}H NMR (400.1 MHz, C_6D_6, 300K): δ 7.41-7.35 (2H, m, H), 7.06-6.99 (3H, m, H), 5.56 (1H, q, H) and 0.95 ppm (12H, d, CH_3 of BPin).

^{11}B NMR (128.38 MHz, C_6D_6, 300K): δ 22.9 ppm (O*B*Pin).

^{13}C NMR (100.62 MHz, C_6D_6, 300K): δ 133.9 (quat Ar-C), 129.5 (Ar-C), 128.7 (Ar-C), 127.9 (Ar-C), 83.7 (C2), 75.1 (C1) and 24.4 ppm (CH_3 of BPin).

Figure S24: Catalytic hydroboration of 2-phenylacetophenone with HBPin using **1t**Li (5 mol%) in C_6D_6

^{1}H NMR spectra

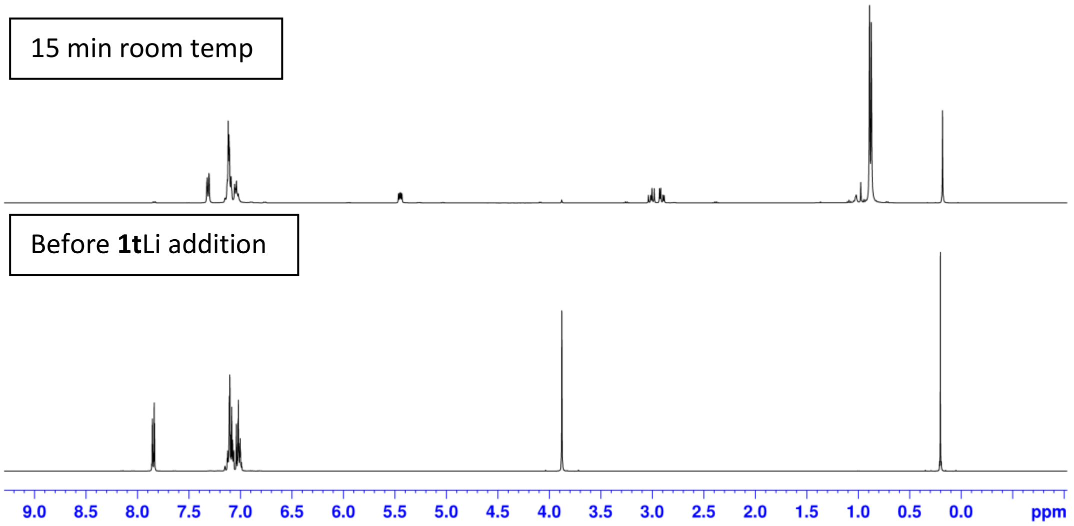

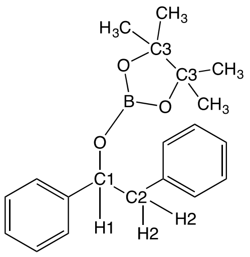

^{1}H NMR (400.1 MHz, C_6D_6, 300K): δ 7.33 (2H, d, Ar-H), 7.16-7.10 (6H, m, Ar-H), 7.09-7.01 (2H, m, Ar-H), 5.47 (1H, q, H1), 3.08-2.89 (2H, m, H2) and 0.89 ppm (12H, d, CH_3 of BPin).

^{11}B NMR (128.38 MHz, C_6D_6, 300K): δ 22.1 ppm (O*B*Pin).

^{13}C NMR (100.62 MHz, C_6D_6, 300K): δ 143.7 (quat Ar-C), 138.7 (quat Ar-C), 130.3 (Ar-C), 128.5 (Ar-C), 128.4 (Ar-C), 127.6 (Ar-C), 126.5 (Ar-C), 126.3 (Ar-C), 82.5 (C3), 78.0 (C1), 46.6 (C2) and 24.5 ppm(CH_3 of BPin).

Figure S25: Catalytic hydroboration of 2-acetylferrocene with HBPin using **1t**Li (5 mol%) in C_6D_6

^{1}H NMR spectra

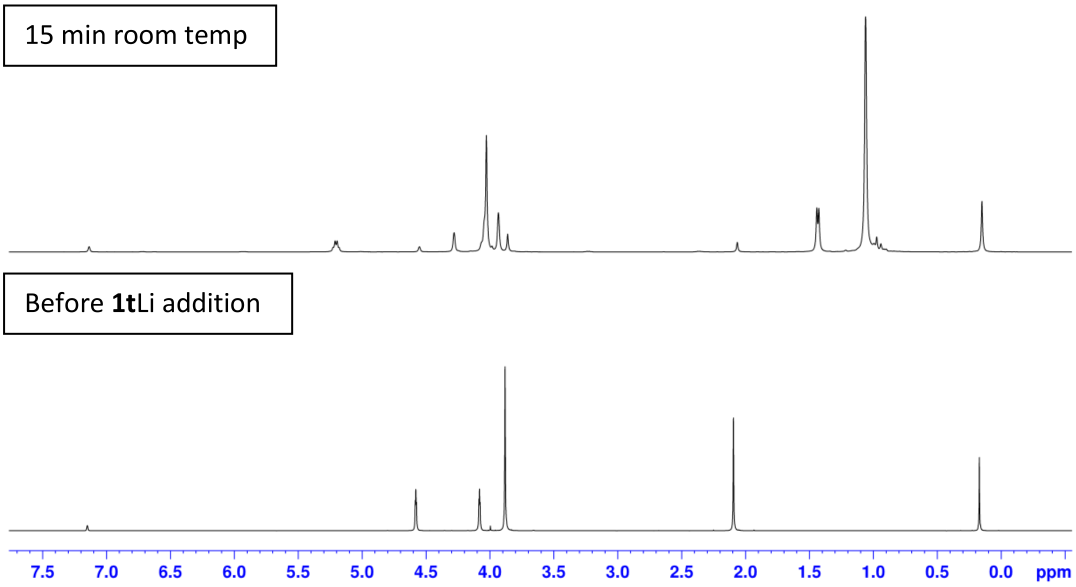

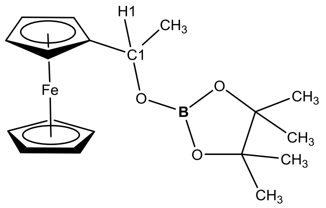

^{1}H NMR (400.1 MHz, C_6D_6, 300K): δ 5.28 (1H, q, H), 4.34 (1H, m, H), 4.08 (1H, m, H), 4.06 (5H, s, Cp ring), 3.97-3.95 (2H, m, H), 1.49 (3H, d, CH_3) and 1.09 ppm (12H, s, CH_3 of BPin).

^{11}B NMR (128.38 MHz, C_6D_6, 300K): δ 22.5 ppm (O*B*Pin).

^{13}C NMR (100.62 MHz, C_6D_6, 300K): δ 92.5 (quat Cp-*C*), 82.6 (C2), 69.3 (Cp ring), 68.0 (Cp-C), 67.5 (Cp-C), 66.1 (Cp-C), 24.8 (CH_3 of BPin) and 24.0 ppm (CH_3).

Figure S26: Catalytic hydroboration of 2-benzoylpyridine with HBPin using **1t**Li (5 mol%) in C_6D_6

^{1}H NMR spectra

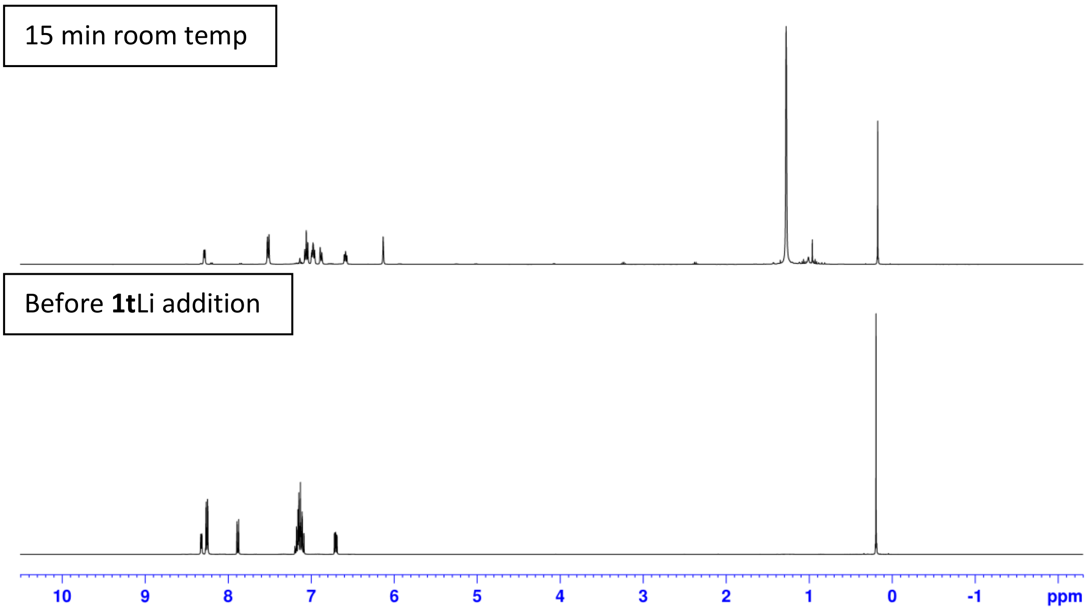

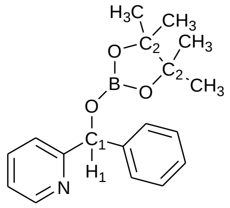

^{1}H NMR (400.1 MHz, C_6D_6, 300K): δ 8.29 (1H, d, Py-H), 7.52 (2H, d, Ar-H), 7.06 (2H, t, Ar-H), 6.98 (2H, m, Py-H), 6.89 (1H, d, Py-H), 6.59 (1H, t, Ar-H), 6.14 (1H, s, H1) and 1.28 ppm (12H, s, CH_3 of BPin).

^{11}B NMR (128.38 MHz, C_6D_6, 300K): δ 16.2 ppm (O*B*Pin).

^{13}C NMR (100.62 MHz, C_6D_6, 300K): δ 162.2 (quat Ar-C), 143.5 (Ar-C), 142.8 (quat Ar-C), 139.1 (Ar-C), 128.6 (Ar-C), 127.8 (Ar-C), 127.1 (Ar-C), 123.1 (Ar-C), 120.5 (Ar-C), 81.0 (C2), 78.7(C1), and 24.9 ppm(CH_3 of BPin).

Figure S27: Catalytic hydroboration of 2,4,6-trimethylacetophenone with HBPin using **1t**Li (5 mol%) in C_6D_6

^{1}H NMR spectra

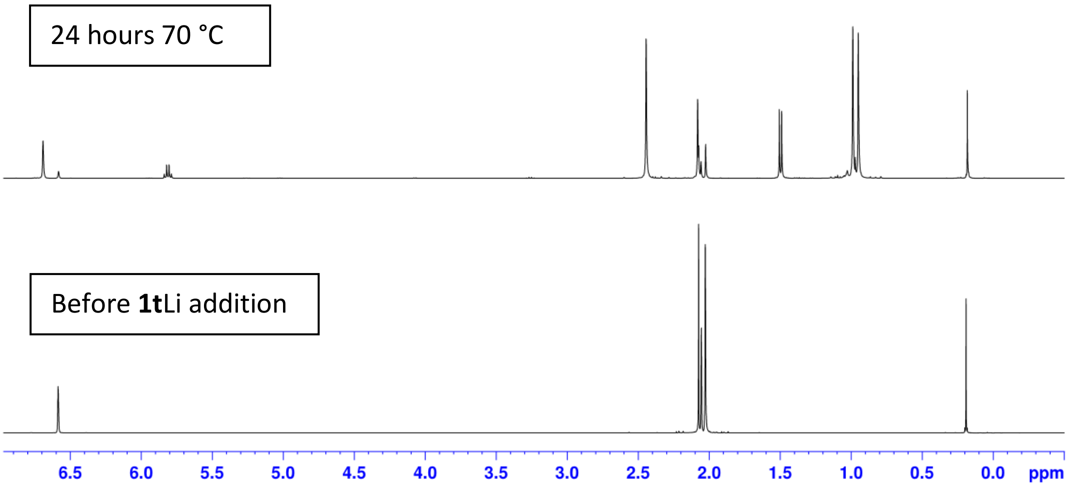

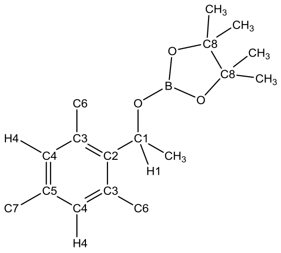

^{1}H NMR (400.1 MHz, C_6D_6, 300K): δ 6.73 (2H, br s, H4), 5.87 (1H, br s, H1), 2.48 (6H, br s, *o*-CH_3), 2.10 (3H, br s, *p*-CH_3), 1.54 (3H, br s, CH_3) and 0.99 ppm (12H, br s, CH_3 of BPin).

^{11}B NMR (128.38 MHz, C_6D_6, 300K): δ 22.4 ppm (OBPin).

^{13}C NMR (100.62 MHz, C_6D_6, 300K): δ 137.4 (quat Ar-C), 136.1 (quat Ar-C), 135.8 (quat Ar-C), 130.3 (Ar-C), 82.4 (C8), 70.3 (C1), 24.7 + 24.5 (CH_3 of BPin), 22.0 (CH_3) and 20.8 ppm (*o*- + *p*-CH_3).

Figure S28: Catalytic hydroboration of 2-butanone with HBPin using **1t**Li (5 mol%) in C_6D_6

^{1}H NMR spectra

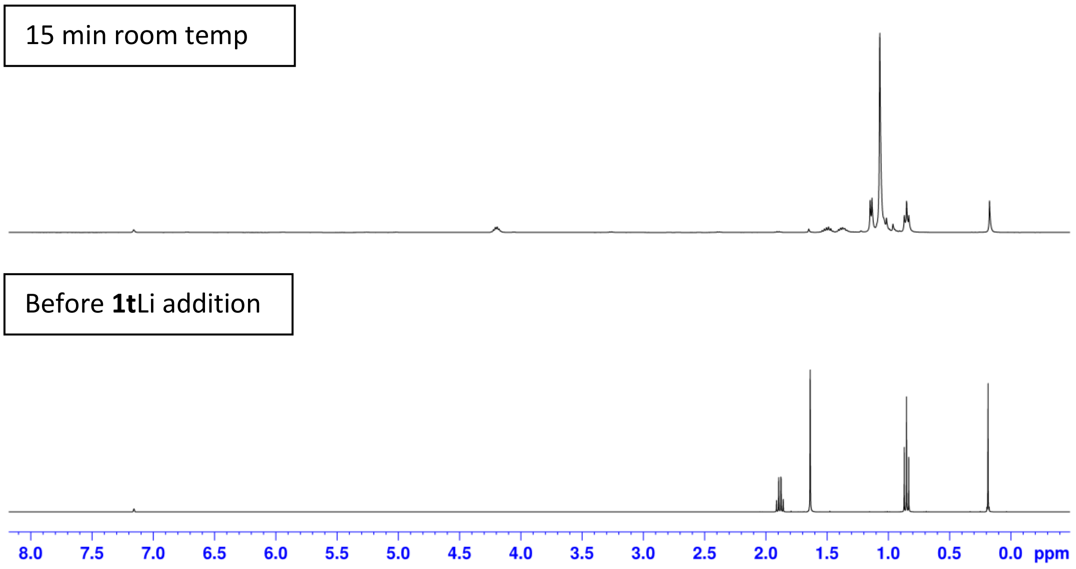

^{1}H NMR (400.1 MHz, C_6D_6, 300K): δ 4.20 (1H, m, CH), 1.50 (1H, m, CH_2), 1.37 (1H, m, CH_2), 1.14 (3H, d, CH_3), 1.07 (12H, s, CH_3 of BPin) and 0.85 ppm (3H, t, CH_3).

^{11}B NMR (128.38 MHz, C_6D_6, 300K): δ 22.3 ppm (OBPin).

^{13}C NMR (100.62 MHz, C_6D_6, 300K): δ 82.2 (quat C BPin), 72.2 (CHOBPin), 31.5 (CH_2), 24.7 (CH_3-BPin), 22.4 (CH_2) and 10.1 ppm (CH_3).

Figure S29: Catalytic hydroboration of di-*t*butylketone with HBPin using **1t**Li (5 mol%) in C_6D_6

^{1}H NMR spectra

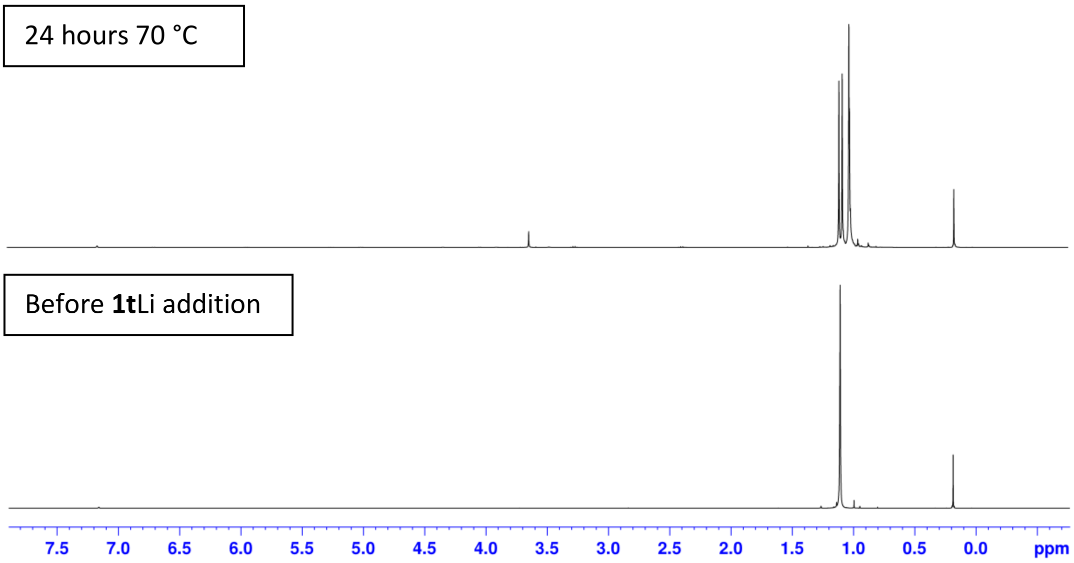

^{1}**H NMR** (400.1 MHz, C_6D_6, 300K): δ 3.64 (1H, s, CH), 1.08 (18H, s, *t*Bu-CH_3) and 1.02 ppm (12H, s, CH_3 of BPin).

^{11}**B NMR** (128.38 MHz, C_6D_6, 300K): δ 22.3 ppm (OBPin).

^{13}**C NMR** (100.62 MHz, C_6D_6, 300K): δ 88.6 (CHOBPin), 82.2 (quat C BPin), 37.7 (q C), 29,0 (CH_3-*t*Bu) and 24.7 ppm (CH_3-BPin).

Figure S30: Catalytic hydroboration of benzaldehyde with HBPin using **1t**Li (1 mol%) in C_6D_6

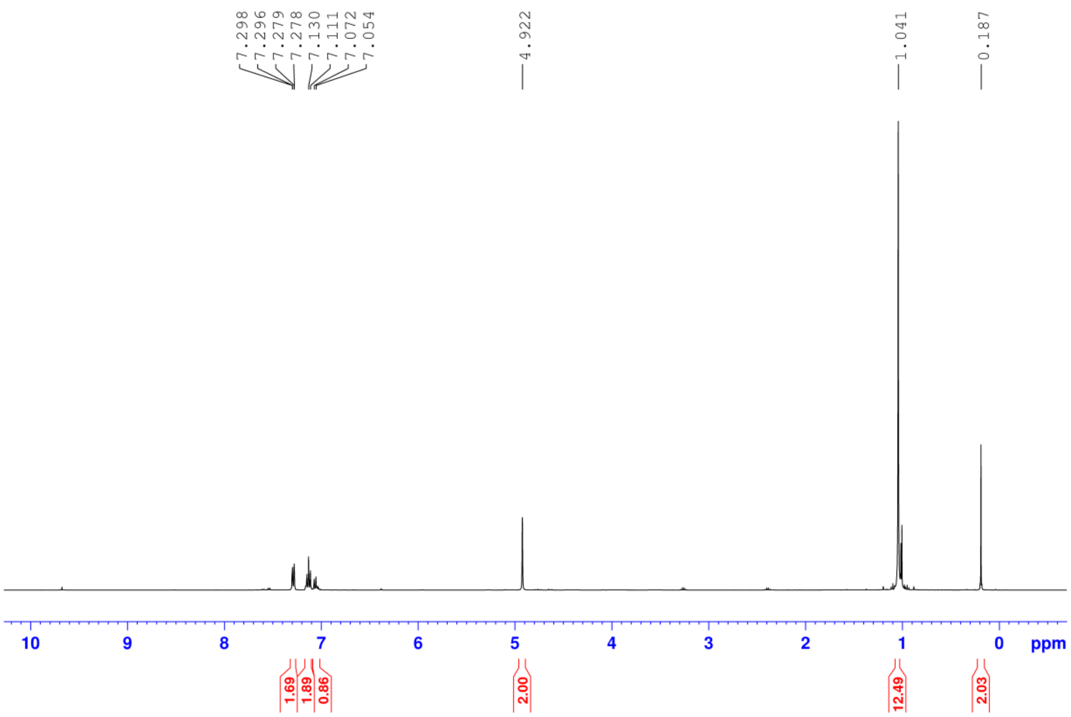

Figure S31: Stoichiometric reaction of **It**Li with HBPin in toluene for 16 h. at room temperature.

^{1}H NMR spectra (d_6-benzene) of **1t**Li and **1t**BPin (aliquot of reaction mixture) showing replacement of Li (lost as LiH) for a BPin unit.

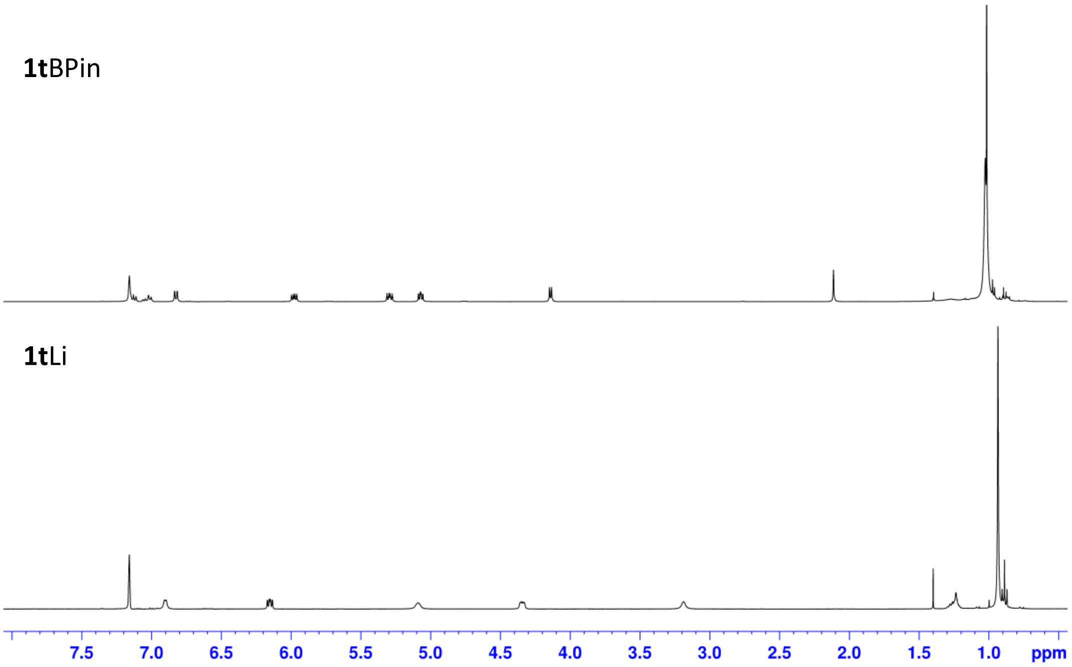

^{7}Li NMR spectra

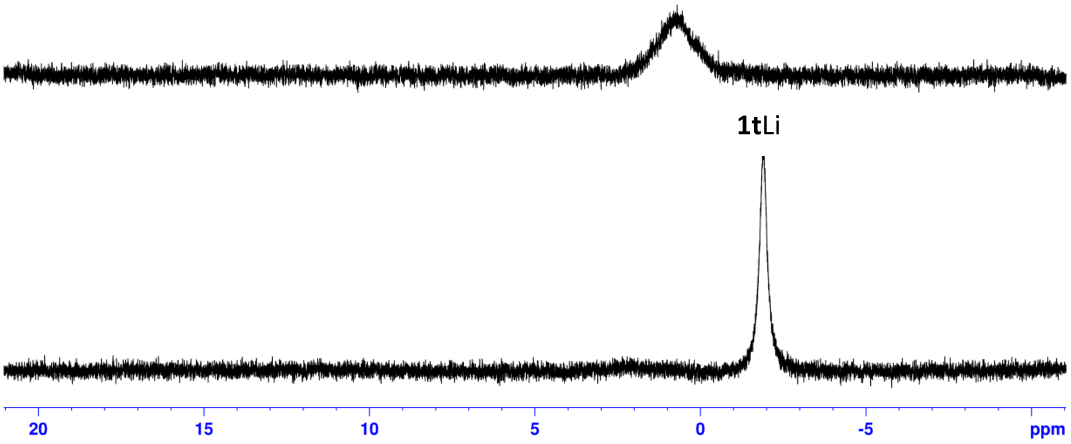

^{11}B NMR spectrum of reaction product showing clear formation of a B-N species (**1t**BPin) due to loss of hydride attached to boron of HBPin.

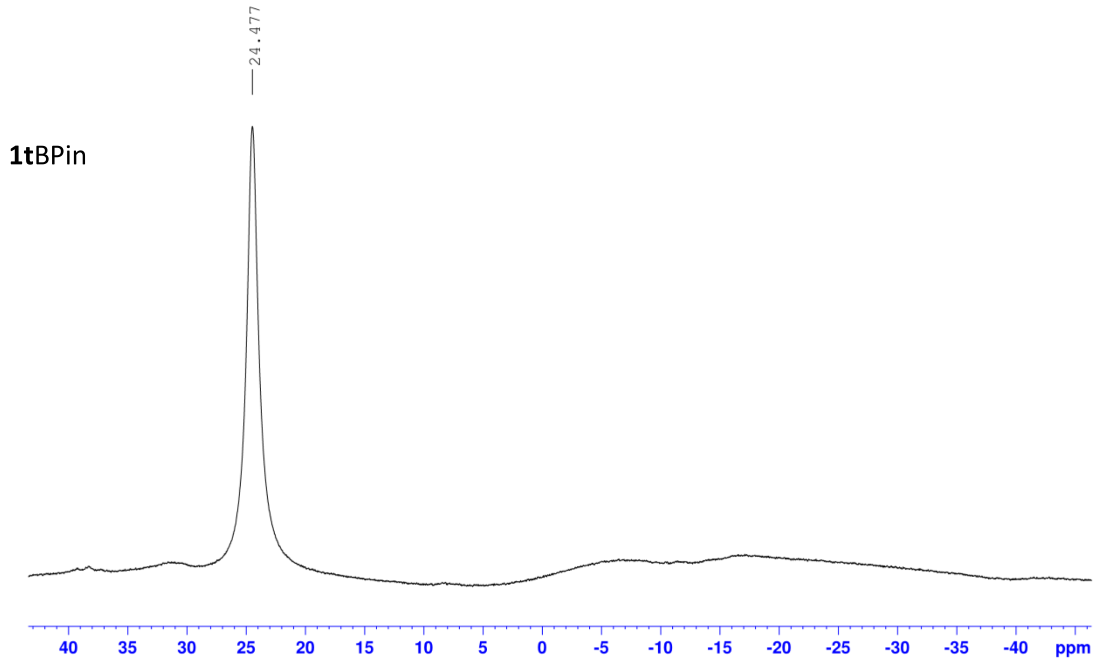

Figure S32: In situ formation of **1t**BPin in toluene and reaction with benzophenone for 16 h. at room temperature. Initially HBPin and **1t**BPin are present. Addition of benzophenone results in slow and incomplete formation of hydroboration product. (Catalytic reaction reaches completion in 1 hour.

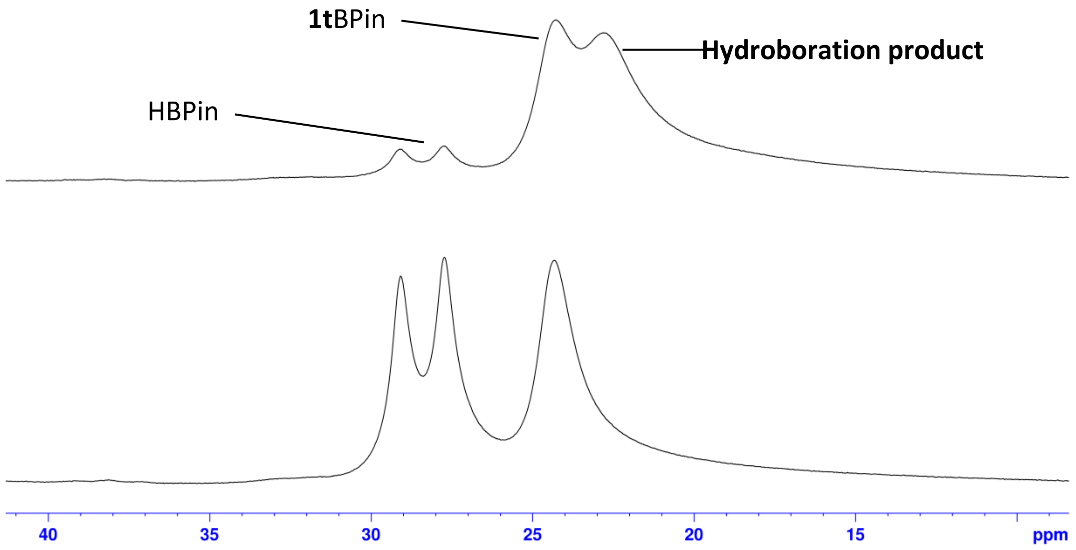

Reaction between pyridine and HBPin: HBPin (0.58 ml 4 mmol) was stirred in excess pyridine (2 mL) for four hours at room temperature. After storage at -30 °C a crop of colourless crystals corresponding to **3** were obtained. Yield 0.447g 58%.

Crystalline **3** decomposed slightly overtime in an inert atmosphere glovebox.

Figure S33: Characterisation of partially decomposed **3**

^{1}H NMR spectra

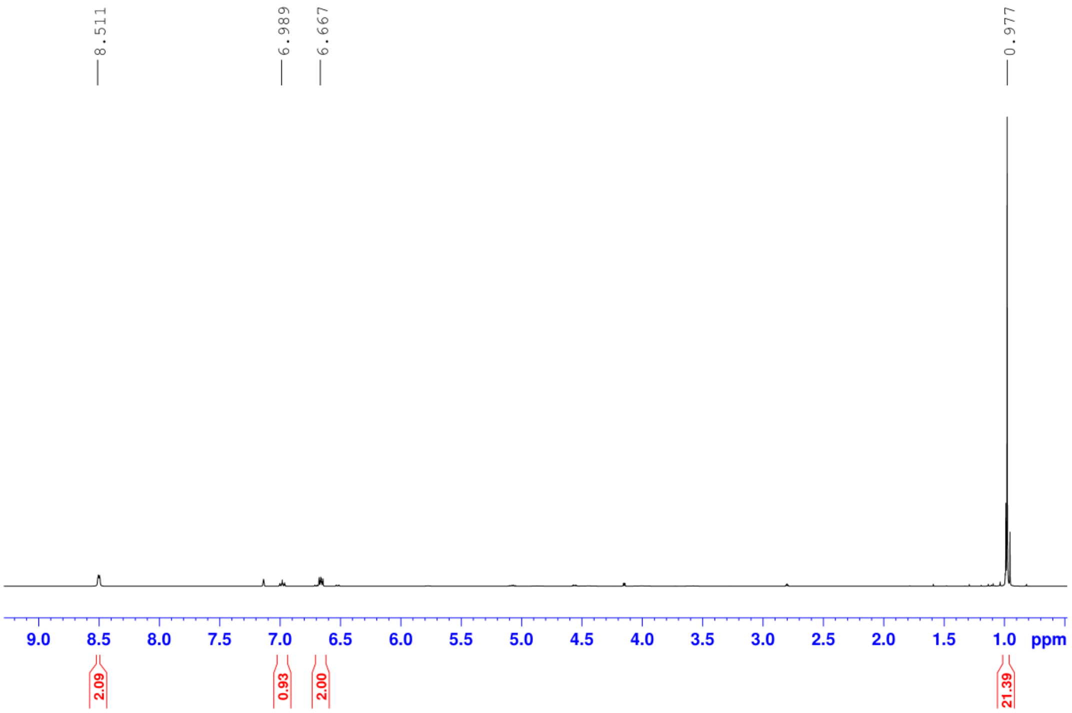

^{11}B NMR spectra

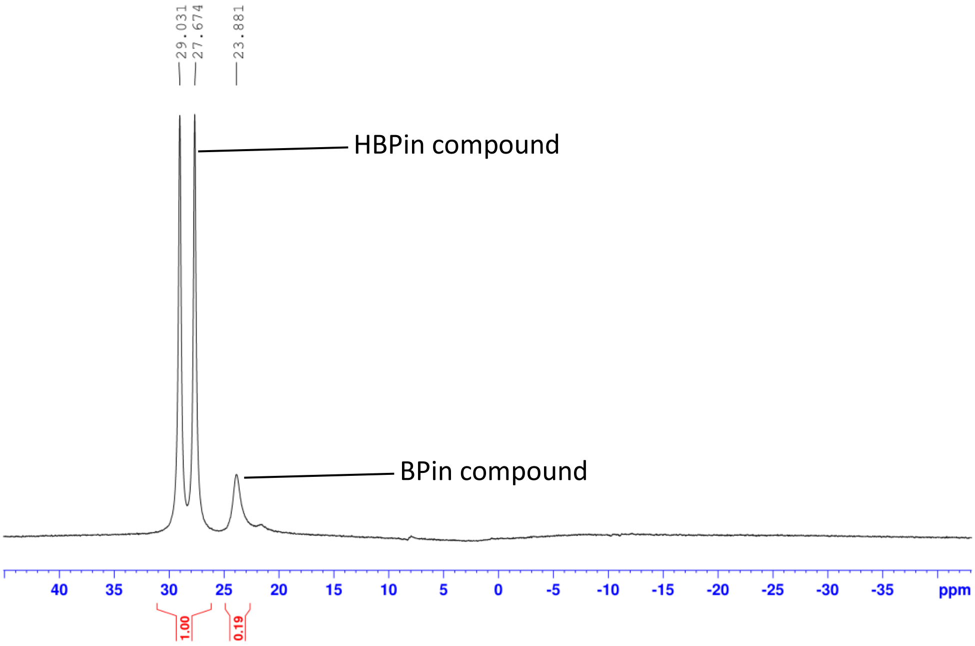

Table S1 Crystallographic data and refinement details for 3

	**3**
Empirical formula	$C_{11}H_{18}BNO_2$
Mol. Mass	207.1
Crystal system	Monoclinic
a/ Å	17.3528(17)
b/ Å	7.5795(4)
c/ Å	11.1117(13)
α	90
β	126.698(16)
γ	90
V/ $Å^3$	1171.8(3)
Z	4
λ/ Å	0.71073
Measured reflections	5806
Unique reflections	2672
R_{int}	0.0337
Observed rflns [I>2σ(I)]	2324
GooF	1.038
R [on *F*, obs rflns only]	0.0402
ωR [on F^2, all data]	0.0884
Largest diff. Peak/hole. e/ $Å^{-3}$	0.21/-0.18

REFERENCES

1. S. D. Robertson, A. R. Kennedy, J. J. Liggat, R. E. Mulvey, *Chem. Commun.* 2015, **51**, 5452–5455.

2. S. A. Orr, A. R. Kennedy, J. J. Liggat, R. McLellan, R. E. Mulvey and S. D. Robertson, *Dalton Trans.* 2016, **45**, 6234-6240.

3. W. Clegg, L. Dunbar, L. Horsburgh and R. E. Mulvey, *Angew*. *Chem*. *Int*. *Ed*. *Engl*., 1996, **35**, 753-755.

4. G. M. Sheldrick, *Acta Crystallogr.* 2007, **A64**, 112-122.

5. O. V. Dolomanov; L. J. Bourhis; R. J. Gildea; J. A. K. Howard; H. Puschmann, *J. Appl. Cryst.* 2009, **42**, 339-341.
